# Long non-coding RNAs: regulators of autophagy and potential biomarkers in therapy resistance and urological cancers

**DOI:** 10.3389/fphar.2024.1442227

**Published:** 2024-10-24

**Authors:** Shizong Wang, Yang Bai, Jie Ma, Liang Qiao, Mingqing Zhang

**Affiliations:** ^1^ Department of Urology, Weifang People’s Hospital, Weifang, Shandong, China; ^2^ Shangdong Provincial Key Laboratory for Prevention and Treatment of Urological Diseases in Medicine and Health, Weifang, Shandong, China

**Keywords:** prostate cancer, non-coding RNAs, autophagy and apoptosis, bladder cancer, renal cancer, biomarkers, therapy resistance

## Abstract

The non-coding RNAs (ncRNAs) comprise a large part of human genome that mainly do not code for proteins. Although ncRNAs were first believed to be non-functional, the more investigations highlighted tthe possibility of ncRNAs in controlling vital biological processes. The length of long non-coding RNAs (lncRNAs) exceeds 200 nucleotidesand can be present in nucleus and cytoplasm. LncRNAs do not translate to proteins and they have been implicated in the regulation of tumorigenesis. On the other hand, One way cells die is by a process called autophagy, which breaks down proteins and other components in the cytoplasm., while the aberrant activation of autophagy allegedly involved in the pathogenesis of diseases. The autophagy exerts anti-cancer activity in pre-cancerous lesions, while it has oncogenic function in advanced stages of cancers. The current overview focuses on the connection between lncRNAs and autophagy in urological cancers is discussed. Notably, one possible role for lncRNAs is as diagnostic and prognostic variablesin urological cancers. The proliferation, metastasis, apoptosis and therapy response in prostate, bladder and renal cancers are regulated by lncRNAs. The changes in autophagy levels can also influence the apoptosis, proliferation and therapy response in urological tumors. Since lncRNAs have modulatory functions, they can affect autophagy mechanism to determine progression of urological cancers.

## Highlights

• LncRNAs are considered as diagnostic, prognostic and therapeutic targets in urological cancers.
• Autophagy is programmed cell death pathway exerting dual function in cancer progresion.
• LncRNAs can change proliferation, metastasis and therapy response in urological cancers.
• The lncRNA-driven regulation of autophagy determines the progression of urological cancers.
• Both lncRNAs and autophagy possess dual function in urological cancers, making it difficult to target them in cancer therapy.

## 1 Introduction

Considering that cancer is an illness that is responsible for a high rate of death and morbidity rate all over the world, researchers have focused their attention over the past few decades on elucidating the function that signaling networks play in the illness. It is well accepted that abnormalities in molecular pathways are the cause of aberrant proliferation and spread of cancer cells (Mohan et al., 2018; Ang et al., 2021). These tumor-promoting molecular pathways, in point of fact, are responsible for the advancement of cancer by activating favorable variables that contribute to cancer survival. Mechanisms that inhibit tumor growth, in contrast, make cancer cells more susceptible to death and stop them from progressing and migrating. Molecular pathways of this kind have been discovered as a result of advancements in sequencing and bioinformatics, and ongoing research has led to the discovery of more new signaling networks that may have an impact on the development or reduction of cancer. The significance of elucidating such molecular pathways is critical because it opens the way for the creation of innovative therapies that are capable of effectively treating cancer. These treatments may be based on the development of genetic tools for the purpose of targeting molecular pathways or about the application of tiny molecules as medications for the purpose of inhibiting the advancement of cancer. In addition, natural compounds produced from plants have shown that they have the ability to target molecular pathways for chemotherapy for cancer. Cancer continues to be a significant obstacle for public health, and there should be an increase in the amount of research committed to gaining a fundamental and clinical knowledge of cancer (Mirzaei et al., 2022a; Paskeh et al., 2022; Wang Y. et al., 2024). It has been considered the gold standard for treating cancer in humans. t to understand the biological foundations of disease and to build plausible molecular therapeutics. Gene therapy is an essential means to attain a possible cure, and it is also one of the most significant ways to get this understanding (Wu Y. et al., 2023). Long noncoding RNAs, often known as LncRNAs, are RNAs that have a transcription length of greater than 200 nucleotides yet cannot code for proteins (Xiao et al., 2018). It is estimated what makes up about 20% of the human genome are genes that code for proteins. Furthermore, eighty percent of the human genome is translated into RNA; however, It is not possible for these RNA transcripts to code for proteins and are thus considered noncoding (Huang et al., 2018; Chen H. et al., 2024). Some elements of the biology of long noncoding RNA (LncRNA) are comparable to that of messenger RNA (mRNA), and RNA polymerase II (Pol II) is able to transcribe most long non-coding RNAs, despite the fact that LncRNA do not encode proteins (Bridges et al., 2021). Even though the amounts of long noncoding RNAs (LncRNAs) are typically lower than those of messenger RNAs (mRNAs), Their expression patterns are more unique to individual tissues. This provides more evidence that long non-coding RNAs (LncRNAs) are still involved in a wide variety of biological processes, such as transcriptional regulation, protein folding, RNA editing, gene modification, and microRNA (miRNA) regulation. (Guo et al., 2020; Si et al., 2021). It is commonly understood that a number of different long noncoding RNAs play a part in controlling cancer’s energy metabolism (Tan et al., 2021). including LUCAT1 (Xing et al., 2021), DUXAP10 (Lin et al., 2021), GAS5 (Ma Y. et al., 2022), TTN-AS1 (Zheng et al., 2021), and others. Moreover, Whether lncRNAs are located in the nucleus or the cytoplasm determines their function. (Ashrafizadeh et al., 2022; Mirzaei et al., 2022b).

LncRNAs have the ability to interact with their targets in either a direct or indirect manner, and they may also act as a scaffold, guide, signal, or decoy to affect proteins, in addition to chromatin and other RNA molecules for the effects (Entezari et al., 2022; Gibb et al., 2011; Moran et al., 2012). LncRNAs have the ability to influence expression of genes simultaneously with those involved in post-transcriptional modifications in the nucleus and the cytoplasm. It should be noted that the role of long non-coding RNAs varies depending on whether they are located in the nucleus or the cytoplasm. Interacting with messenger RNAs (mRNAs), lncRNAs that are found in the cytoplasm are responsible for regulating gene expression at both the translational and post-transcriptional stages. In addition, long noncoding RNAs have the ability to interact with microRNAs by performing the function of competitive endogenous RNAs (ceRNAs) and lowering the production of miRNAs. On the other hand, long noncoding RNAs that are found in the nucleus have a distinct function and are able to associate with proteins and transcription factors; participate in DNA methylation; modify histones; remodel chromatin (Lu et al., 2021; Tang et al., 2023).

## 2 LncRNAs in oncology

Within the system that governs epigenetic regulation, lncRNAs play an essential function (Alharthi et al., 2024). By having an effect on the structure of chromatin (Xiang et al., 2014; Postepska-Igielska et al., 2015; Wang et al., 2011), the modification of histones (Sati et al., 2012; Grote et al., 2013), alternative transcription (Gonzalez et al., 2015), the suppression of X-chromosomes (Froberg et al., 2013), and the reimbursement of dosage (Samata and Akhtar, 2018). In addition to their ability to influence expression of genes during transcription, epigenetic modifications, and the post-transcriptional phase, lncRNAs have been linked to a wide range of cellular functions and molecular signaling cascades (Liz and Esteller, 2016; Jiang et al., 2021). Despite the fact that they are unable to produce translation proteins, lncRNAs are nevertheless able to make a contribution to affect transcription by manipulating transcription factors, enhancers, and initiators (Engreitz et al., 2016; Kim et al., 2010; Li W. et al., 2016). Furthermore, long noncoding RNAs have the ability to affect post-transcriptional changes in a manner that helps to preserve messenger RNAs and serves as a precursor for small noncoding RNAs (Jalali et al., 2012; Song et al., 2018; Yang et al., 2014). Alternatively, lncRNA) can be seen as contending for endogenous RNAs (ceRNAs), which compete with sponge microRNAs such that downstream gene targets can be addressed (Sen et al., 2014; Liang et al., 2015; Han et al., 2020; Thomson and Dinger, 2016; Jarlstad, 2021; Hussain et al., 2023; Xie et al., 2023). Several long non-coding RNAs have been associated with alterations that are associated with cancer. These lncRNAs also perform crucial activities in regulatory genes, which cause them to influence a variety of elements of the cellular homeostasis, which encompasses development, propagation, migration, and genetic integrity (Huarte, 2015). Evidence suggests that certain LncRNAs play a part in the stemness of tumors by controlling the establishment of transcription variables associated to malignant stem cells (Chen et al., 2017; Liu B. et al., 2021). For example, the long noncoding RNA CCAT2, This represents an overexpressed gene in CRC, has the ability to activate the Wnt signaling cascade and regulate c-Myc transcription to improve tumor invasion and spread. (Ling et al., 2013). Given that c-Myc is responsible for the post-transcriptional activity, the long noncoding RNA known as CCAT1 has the potential to accelerate the progression of gastric cancer (GC) (Yang et al., 2013; Alharbi et al., 2022).

A large number of lncRNAs have recently been linked to cancer initiation and progression. It is possible for them to function act as either tumor suppressors or oncogenes (Martens-Uzunova et al., 2014). Many different forms of cancer have been linked to a large number of lncRNAs., including malignancies of the breast, ovary, pancreas, prostate, and other organs. TUG1, NEAT1,HOTAIR, and CCAT1are examples of lncRNAs that might potentially cause cancer. On the other hand, DANCR, GAS5, MALAT1, and UCA1 are examples of lncRNAs that could potentially inhibit cancer. These long noncoding RNAs have an effect on critical pathways that are related with the growth and spread of cancer, as well as EMT and MDR (Bhan et al., 2017; Braga et al., 2020; Arriaga-Canon et al., 2023; Adnane et al., 2022; Connerty et al., 2020). In addition, Prolonged noncoding RNAs (lncRNAs) have been demonstrated to play a role in significant regulatory actions inside the cell and have been connected to a variety of diseases, not the least of which is cancer. The medicinal relevance of long noncoding RNAs (lncRNAs) for use as diagnostic, therapeutic, and prognostic biological markers is now being researched. Additionally, lncRNA-based diagnostics and therapies are currently being developed in order to enhance personal healthcare and standard of living (Zhang and Tang, 2018; Bhat et al., 2023; Naderi-Meshkin et al., 2019; Hanly et al., 2018). Recent research has shown that long noncoding RNAs (lncRNAs) also play an important part in the molecular response of tumors (MRD) (Figure 4) (Majidinia and Yousefi, 2016). In light of these findings, it is possible that they might be utilized as target therapeutics in the battle against cancer.

**FIGURE 1:**
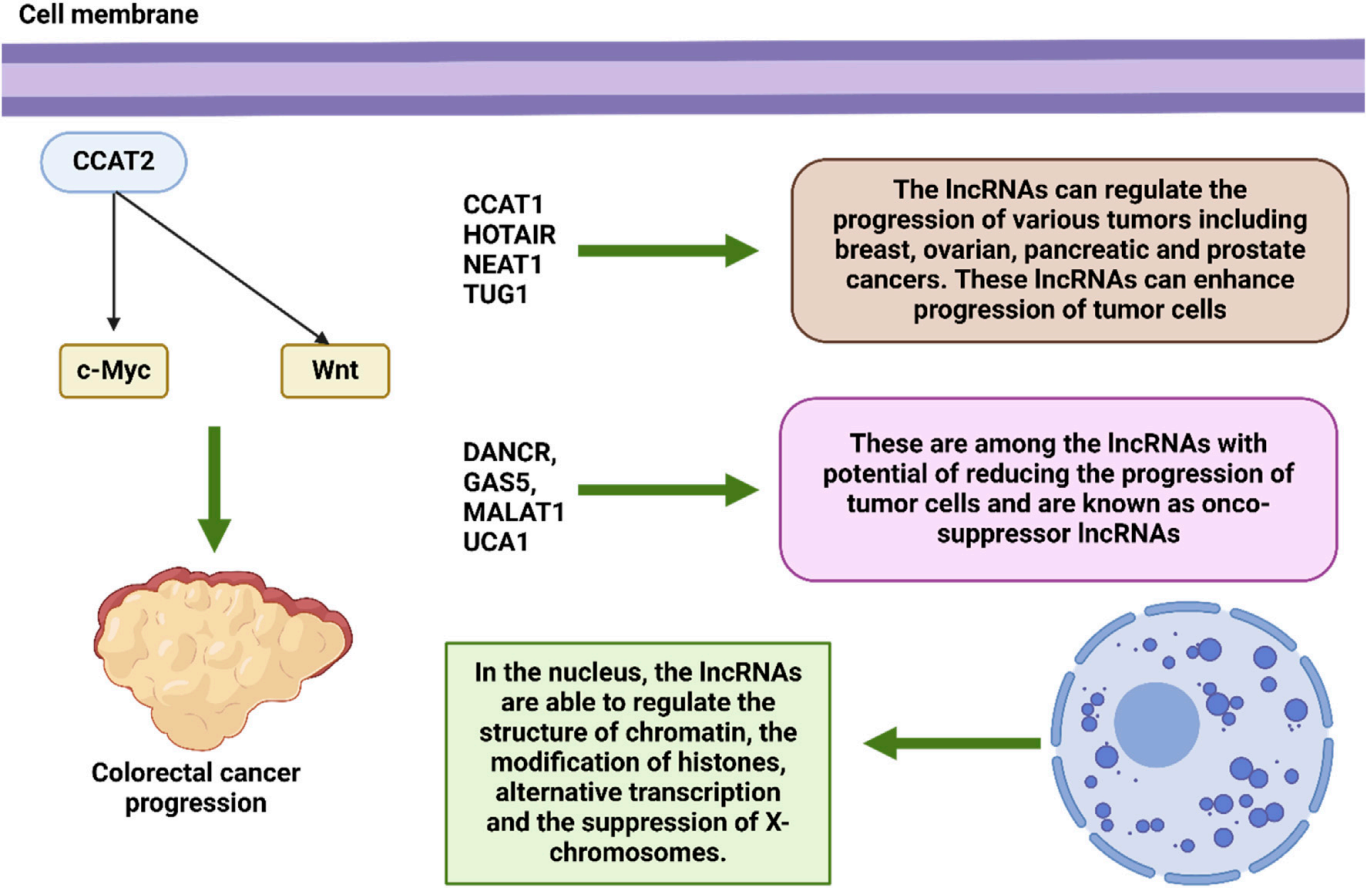
The mechanism of action of lncRNAs in the regulation of tumorigenesis.

The deregulation and functional involvement of lncRNAs in cancer provide novel opportunities for expanding the existing diagnostic and therapeutic toolbox for this complex disease (Begolli et al., 2019). Regarding diagnosis, the discovery of circulating oncogenic lncRNAs in tumor-derived exosomes, coupled with their specific spatiotemporal activation, currently holds great promise for the development of highly specific diagnostic markers (Xu et al., 2018; Kim et al., 2015). Exosomes are a group of extracellular vesicles that arise when intermediate endosomal compartments, known as multivesicular bodies (MVBs), fuse with the plasma membrane to release their contents (Edgar, 2016; Harding et al., 1983). Exosomes function as vehicles of cell-to-cell communication and have been implicated in various diseases, including cancer (Edgar, 2016; Milane et al., 2015). These vesicles, ranging from 30 to 100 nm in size, contain a wide assortment of molecular cargos such as proteins, lipids, and nucleic acids, including miRNAs, mRNAs, and lncRNAs (Shurtleff et al., 2017; Kogure et al., 2013). Several lncRNAs that epigenetically regulate cancer cells through various mechanisms are also part of the exosomal cargo secreted from tumors. Examples of lncRNAs that interact with the epigenetic machinery and have been detected in exosomes include MEG3 and HOTAIR, which are secreted specifically from cervical tumors but not from their normal counterparts, offering opportunities for developing RNA-centric diagnostic approaches (Zhang J. et al., 2016). Other examples of lncRNAs secreted from tumor exosomes include LUCAT1 and PVT1 in exosomes of liver cancer (Gramantieri et al., 2018; Yu et al., 2016). In contrast, secreted exosomes from normal intestines carry significantly higher levels of HOTTIP than their colon cancer counterparts, providing novel opportunities for monitoring disease onset (Oehme et al., 2019). Interestingly, exosomal packaging appears to increase the stability (and therefore detection threshold) of NEAT1 and certain other lncRNAs compared with their intracellular levels (Gezer et al., 2014). Evidence suggests that lncRNAs, apart from being secreted, can also exert significant control over the production of exosomes in cancer. For instance, lncRNA-APC1, which is downregulated in colorectal carcinoma cells (CRCs) due to mutations in its master regulator APC, is a tumor-suppressor transcript that inhibits angiogenesis, proliferation, and migration of cancer cells. With exosomes playing a vital role in the induction of angiogenesis in CRCs, it has been shown that lncRNA-APC1 exerts its function by decreasing the stability of Rab5b mRNA, an important regulator of the exosome production process, ultimately reducing overall exosome production (Wang FW. et al., 2019). Figure 1 demonstrates the potential of lncRNAs in the regulation of carcinogenesis.

## 3 Urological cancers: An overview

### 3.1 Prostate cancer

There are around 180,000 new instances of prostate cancer diagnosed on an annual basis in the USA, which is equivalent to approximately over 20% of newly diagnosed cancer cases (Siegel et al., 2018; Howard et al., 2019). Among male-specific malignancies, prostate cancer ranks high. Biological processes of drug resistance eventually limit therapies for metastatic sickness, notwithstanding the efficacy of prostatectomy or radiation therapy for early stage localized prostate cancer. This is the case even if these treatments are often effective. Orgasmic suppression treatment (ADT), upon which the androgen receptor pathway is focused, is the primary treatment dealing with men who have progressed to advanced stages of prostate cancer (Kirby et al., 2011; Huggins and Hodges, 1941). Being an illness, prostate cancer is the reason behind this. that is driven by androgens. Despite the fact that ADT is initially beneficial (Siegel et al., 2018; Ferlay et al., 2013), the vast majority of patients eventually develop resistance to the treatment, CRPC, which stands for castration-resistant prostate cancer, and androgen-independent prostate cancer. Crbazitaxel, sipuleucel-T, docetaxel, enzalutamide, radium-223, and abiraterone are some of the treatment choices that are available for metastatic CRPC for patients who have undergone ADT before. In addition, studies, including the one that we conducted, have shown that early combination treatment with ADT and docetaxel or ADT plus Abiraterone is beneficial to survival for some patients who had metastatic cancer (Sweeney et al., 2015; James et al., 2016; Fizazi et al., 2017; James et al., 2017). Despite the availability of all treatment options, metastatic CRPC continues to be incurable, and eventually medication resistance will emerge (Amaral et al., 2012; Chandrasekar et al., 2015). Upregulation downstream of AR, alterations to AR splice variants and co-regulatory proteins, alterations to AR gene amplifications and mutations, and changes to the expression of AR steroid-generating enzymes are some of the processes that have been investigated as potential contributors to challenges in targeting the androgen receptor axis (Nakazawa et al., 2017).

Using morphologic criteria, the Gleason total score (Gleason, International Cancer Control Union) is used to describe the pathologic categorization of prostate cancer (Logothetis et al., 2013). This score is based on characteristics of the prostate. Regarding prostate cancer, it is the single most important indicator of prognosis. and the Gleason score is the primary way for classifying the tissue of prostate cancer (Gleason, 1966; Gleason and Mellinger, 1974). It is possible that intensive therapies are required if the Gleason score is high since it indicates that the development will be more fast. The Gleason score, on the other hand, does not offer any information on the choice of therapy. As a consequence of this, patients are presently classified in accordance with their current treatment state or clinical stage (for example, in the presence or absence of bone metastases, androgen ablation therapy resistance; chemotherapy efficacy). Through the use of this framework, patients that have similar prognoses are categorized (Ryan et al., 2006; McKenney et al., 2011; Ou et al., 2024). Therefore, the design of clinical trials is now determined by these parameters. This technique, on the other hand, lacks the molecular basis necessary to direct the proper molecularly targeted medication sequences or combinations. In addition, the current prostate cancer progression model does not take into consideration the finding that the state of cancer advancement is the determining factor in the efficacy of a particular medicine of choice. For instance, androgen ablation, chemotherapy-free, is more effective when administered at an earlier stage in the evolution of prostate cancer (Gravis et al., 2013). There is a paradoxical relationship between the latter phases of prostate cancer growth and the effectiveness of treatment (Efstathiou et al., 2010; Efstathiou and Logothetis, 2010; Millikan et al., 2008). The fact that the response to therapies varies depending on the stage of the disease suggests that prostate cancer goes through a progression that creates multiple states as the disease progresses. Additionally, the progression of prostate cancer is site-specific. which means that the prostate and bone are two favored locations of cancer that is either persistent or recurrent. Despite the fact that lymph nodes can potentially get affected by prostate cancer, these metastases are often not resistant to treatment. Based on these data, it appears that prostate cancer has a distinct association with the particular microenvironment that exists inside the prostate and bone (Loberg et al., 2005; Logothetis and Lin, 2005). Although every one of these characteristics is important from a therapeutic standpoint, they do not serve as a point of reference for choosing a therapy.

### 3.2 Bladder cancer

It is estimated that the number of newly diagnosed cases of bladder cancer in 2018 reached 549,393, making it the biggest cause of death throughout the globe (Mirzaei et al., 2022c; Bray et al., 2018). There are two subtypes of bladder cancer, which are referred to as non-muscle invasive bladder cancer (NMIBC) and muscle-invasive balder cancer (MIBC). Both of these subtypes have different molecular patterns. It is still a cause of mortality, despite the fact that there have been advancements in the field of biology and medicine for the treatment and diagnosis of breast cancer. In an effort to enhance the prognosis and overall survival rate of patients with breast cancer, there have been efforts made to create clinical treatments. The advancements that have been made in the field of bioinformatics and large-scale gene expression have led to the introduction of molecular profiles as a basis for diagnosing breast cancer (Tran et al., 2021; Sim et al., 2019; Deng et al., 2024). There is a significant amount of application of surgery, chemotherapy, radiation, and immunotherapy for patients with breast cancer; yet, these patients continue to have a poor prognosis, and their overall survival rate over a period of 5 years is low (Parizi et al., 2020; Ashrafizadeh et al., 2020). Regarding the origin, the majority of BC originates from the urothelial layer, and this particular kind of BC is prevalent in the United States and Europe. On the other hand, BC in its non-epithelial variant is prevalent in other parts of the world due to the presence of persistent schistosomiasis (Rhea et al., 2021). Both nuclear anaplasia and architectural changes are taken into consideration when determining the BC grade (Epstein et al., 1998). The fact that individuals with NMIBC who are having therapy may have a return of the disease is something that should be mentioned since it demonstrates the significance of follow-up and subsequent medications. When compared to Migrant-inducible B-cells, of which the invasion and metastatic rates are quite high, which results in a high mortality rate among patients, recurrence is a growing concern among women whose breast cancer has not spread to the muscle (NMIBC) (Wang Y. et al., 2020). The high prevalence of gene mutations that are associated with breast cancer is one of the most intriguing aspects of this kind of cancer. This rate is equivalent to that of other types of cancer, such as lung and skin cancers, and have found that the gene encodes the enzyme TERT, which is involved in telomerase reverse transcription. is the most prevalent mutation that is identified in individuals with breast cancer (up to 70–80 percent) (Lawrence et al., 2013; Alexandrov et al., 2013; Rachakonda et al., 2013; Leão et al., 2019; Kurtis et al., 2016; Allory et al., 2014). The identification of molecular components that contribute to the initiation of breast cancer is thus of interest. Recent investigations have concentrated on identifying the elements that are responsible for the development of breast cancer and the therapeutic targeting of those factors. In addition, various molecular routes that are downregulated in breast cancer, and increasing the expression of these pathways is essential for the efficient elimination of cancer (Du et al., 2022; Wu et al., 2020; Shen et al., 2020; Liu et al., 2020; Li Y. et al., 2020).

### 3.3 Renal cancer

It is the 10th most prevalent cancer in the world (Grange et al., 2019; Petejova and Martinek, 2016) and the third most common urogenital malignancy (Williamson et al., 2019; Taneja and Williamson, 2018). Renal cell carcinoma (RCC) is responsible for around three percent of all adult cancers. The colorectal cancer (RCC) is one of the malignancies that is growing at the quickest rate, and it is anticipated that this trend will continue over the next 20 years (Znaor et al., 2015). Males have a greater risk of developing RCC. The majority of renal cell carcinomas are clear-cell varieties. accounting for up to 80 percent of all new instances of RCC. This is despite the fact that there are other histological subtypes of RCC that have been discovered. Histologically speaking, clear-cell rheumatoid carcinoma is distinguished through the existence of cancer cells with cytoplasm that is visible to the naked eye. This is because of cholesterol esters, phospholipids, glycogen, and a cell membrane’s accumulation that is well defined (Rini et al., 2009). Papillary carcinoma, chromophobe reticulocellular carcinoma, and collecting-duct carcinoma are the additional subtypes. The best prognosis is for chromophobe renal cell carcinoma., is fairly uncommon (Patard et al., 2005), but papillary RCC, which accounts for fifteen percent of all cases of RCC, is the most common kind of cancer in kidney transplant patients.

**FIGURE 2:**
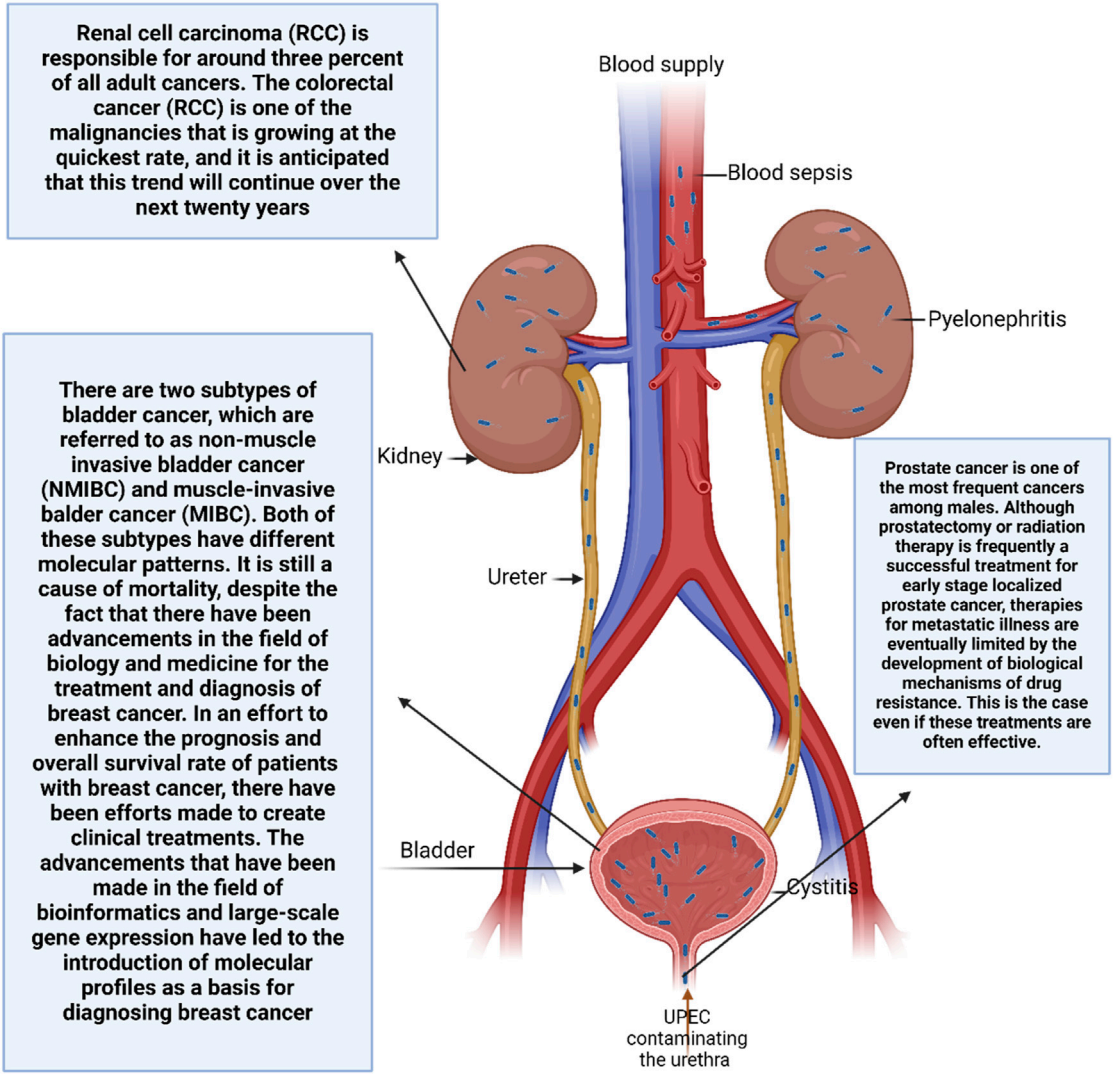
An overview of major urological cancers.

It is known that a large number of genetic mutations have a role in the development and course of RCC, and the discovery of these mutations would help to improved diagnostics and prognoses (Schmidt and Linehan, 2016). One of the most important aspects of the process of developing new particular anti-cancer therapy techniques is this. The inactivation of the tumor suppressor von Hippel-Lindau (VHL) which can be caused by mutations, loss of heterozygosity, or promoter hypermethylation is the most frequent genetic aberration and was the first to be documented (Kim et al., 2018). Additionally, A multi-protein complex known as the E3 ubiquitin ligase includes the VHL protein. that is responsible for regulating the breakdown of proteins by proteasomes (Maxwell et al., 1999). As a result of an impairment in VHL, there is an increase in the expression of hypoxia inducible factors (HIF)-1α and 2α. These HIFs homodimerize and increase the production of proteins that promote angiogenesis, particularly platelet-derived growth factor (PDGF) and vascular endothelial growth factor (VEGF). (Brauch et al., 2000; Courtney and Choueiri, 2010). In particular, endothelial cell proliferation is enhanced by activating pathways linked to VEFG. as well as their migration and survival. The clear-cell RCC subtype is the most common location for this genetic mutation to be found. However, deactivating VHL is insufficient on its own to instigate the development of RCC (Petejova and Martinek, 2016; Brauch et al., 2000). The genes SET domain containing 2, BRCA1-related protein-1, lysine K-specific demethylase 6A, and PBRM1; the SWI/SNF chromatin-remodeling complex gene; are some of the other mutations that have been characterized as contributing to the onset and advancement of recurrent cervical cancer. Twelve. Furthermore, it has been demonstrated that the mammalian target of rapamycin (mTOR) pathway, which plays a role in the control of cell proliferation in response to hypoxia, is considerably elevated in RCC (Rausch et al., 2019). Studies on the patterns of microRNA (miRNA) expression in RCC tissue specimens have been conducted somewhat recently., and the results have shown that there is an overexpression of miRNAs where tumor-suppressors are targeted, whereas microRNAs that specifically target cancer genes are downregulated (Grange et al., 2014; Mytsyk et al., 2018). Deregulated microRNAs have an effect on critical molecules that are involved in the advancement of RCC, including HIF,mTOR, VEGF, VHL, and PTEN (Moch et al., 2015). The high risk of metastasis and the difficulty in diagnosis are two of the factors that contribute to the poor prognosis associated with RCC. In actuality, more than sixty percent of RCC are discovered by accident. It is estimated that around twenty to thirty percent of all patients already have illness that has spread throughout the body when diagnosed (Petejova and Martinek, 2016), and approximately thirty percent of patients who have been treated for localized RCC experience a recurrence in distant locations (Ahrens et al., 2019; Barata and Rini, 2017). This is despite the fact that imaging methods have been improving. There is a survival rate of fewer than 10% for individuals who have metastatic RCC (Cairns, 2011; Graves et al., 2013). This indicates that the prognosis for these patients is quite bad. The insufficient elimination of tumor cells is one of the variables that contribute to the failure of therapy, and this may be the result of the heterogeneity of the treated cells. Particularly, Researchers are becoming increasingly interested in the limited number of cancer stem cells (CSCs) because they are thought to be the main culprits behind tumor recurrence and medication resistance. (Figure 2) (Corro and Moch, 2018; Bussolati et al., 2008). This is because CSCs are the progenitor cells of cancer.

## 4 LncRNAs in prostate cancer

### 4.1 LncRNAs in prostate cancer progression

The little noncoding RNA known as CCAT1 is thought to be a tumor-promoting agent, and its significance in a variety of malignancies has been examined (Mirzaei et al., 2022b). The protein known as CCAT1 has been shown to promote the development of endometrial cancer, while simultaneously reducing the estrogen receptor-alpha (ERν) expression level and the molecular networks associated with it (Treeck et al., 2020). with example, CCAT1 has the ability to manage miRNA-138-5p and miRNA-181a-5p in pancreatic and colorectal malignancies through respectively, with the purpose of altering progression (Shang et al., 2020). This is supported by the growing body of data that supports the regulatory influence of the long noncoding RNA CCAT1 on the expression of miRNA in various malignancies. Within prostate tumors, CCAT1 is responsible for promoting tumor growth and development. This explains why CCAT1 cytoplasmically interacts with miRNA-28-5p, which results in a decrease in the amount of expression, and this interaction lays the path for the advancement of prostate cancer (You et al., 2019). It is important to note that various upstream mediators in prostate cancer can have an effect on long noncoding RNAs (lncRNAs) in order to modulate their regulatory effects on microRNAs. This kind of behavior takes place with the long noncoding RNA FOXP4-AS1, which blocks cell death in prostate tumors. and dramatically boosts proliferation and metastasis. Paired box 5 (PAX5) has the ability to stimulate the production of FOXP4-AS1, which then acts as a ceRNA for miRNA-3184-5p. This ultimately results in the enhancement of FOXP4 expression and its post-transcriptional regulation, which is beneficial to the advancement of prostate cancer (Wu et al., 2019). It is necessary to conduct further experiments in order to investigate the complex molecular pathways that have emerged as a result of the regulation of long noncoding RNAs (lncRNAs) by upstream mediators and their interaction with the production of microRNAs. The long noncoding RNA LINC00665 is a newly discovered component in cancer that plays an important part in the regulation of a variety of cellular pathways. An additional experiment underlines the fact that increased expression of LINC00665 is associated with a worse prognosis for men with prostate cancer. (Eke et al., 2021). This is despite the fact that there is data suggesting that LINC00665 suppresses the growth of glioma through STAU1-mediated mRNA degradation (Ruan et al., 2020).

As a result, LINC00665 is capable of playing a role in the development of tumors in prostate cancer and may be considered a tool for diagnosis and prediction. In prostate cancer, STaphylococcal nuclease and Tudor domain containing 1 (SND1) overexpression is associated with the growth of the disease, and the expression of SND1 is downregulated by miRNA-1224-5p, which is responsible for stopping the advancement of cancer. Through the process of sponging miRNA-1224-5p and the consequent overexpression of SND1, it has been revealed that LINC00665 is responsible for the enhancement of tumor propagation, proliferation, and metastasis (Chen W. et al., 2020). As a result, It is well-known that lncRNAs can promote tumors by targeting microRNAs, which are a type of lncRNA. have the ability to influence their production by sponging in the process of influencing the growth of prostate cancer (Wu et al., 2019; Zhang et al., 2020). In a variety of malignancies, the long noncoding RNA SNHG4 acts as an oncogenic component. There is a multi-targeting capability of the long noncoding RNA SNHG4, which also influences a variety of pathways that promote tumor malignancy. By avoiding the arrest of the cell cycle and enhancing proliferation and spread of tumor cells, In gastric cancer, RRM2 is upregulated through miRNA-204-5p when SNHG4 is overexpressed. (Cheng et al., 2021). This action is taken in order to prevent cell cycle arrest. SNHG4, a long noncoding RNA, has a role in the process of boosting the metastasis of gastric tumor cells by the activation of EMT through the sponging of miRNA-204-5p (Wang et al., 2021). Additionally, it plays a role in the immune evasion of cancer cells (Zhou et al., 2021). When prostate cancer is present, the identical event takes place, and an upstream mediator known as SP1 causes SNHG4 to acquire an increased level of expression. Then, SNHG4 stimulates the production of ZIC5 by the sponging of miRNA-377, which has the effect of increasing the malignant behavior of tumor cells and enhancing their survival (Wang ZY. et al., 2020). In the event that a tumor-promoting long noncoding RNA is identified, the most effective methodology for decreasing the rate of prostate cancer’s advancement is to knock it down. In the case of prostate cancer, for example, inhibiting the long noncoding RNA TUG1 is advantageous in terms of suppressing the disease and increasing radiosensitivity through the overexpression of miRNA-139-5p and the consequent overexpression of SMC1A (Xiu et al., 2020). In order to overcome the propensity of prostate tumor cells to mediate chemoresistance, further research is required (Quintanal-Villalonga et al., 2020). Because of the relationship between lncRNA and miRNA, treatment resistance in prostate tumors is determined. An increase in transcript levels of the long noncoding RNA the NEAT1 causes a resistance to docetaxel in prostate tumors. Increasing the expression of miRNA-204-5p and miRNA-34a-5p, which are both downregulated in prostate cancer, brings to an increase in chemosensitivity by inhibiting the expression of ACSL4. Because it acts as an upstream mediator, the long noncoding RNA NEAT1 brings down the levels of miRNA-204-5p and miRNA-34a-5p, which in turn raises the expression of ACSL4, which ultimately results in prostate tumor cells being resistant to docetaxel (Li X. et al., 2020).

Furthermore, additional lncRNAs that prostate cancer tissues, which are dysregulated, add to the advancement of the disease through processes that are completely distinct (Mitobe et al., 2018). HOX transcript antisense RNA, also known as HOTAIR, is a kind of long noncoding RNA that has been extensively studied and has been shown to be tumorigenic. The antisense strand of the HOXC gene cluster is where the transcription of HOTAIR takes place. According to the first findings of Rinn et al. (Rinn et al., 2007), PRC2-mediated histone H3 lysine-27 trimethylation at the HOXD gene locus requires HOTAIR. This interaction with PRC2 was determined to be crucial. The opposite is true, according to a paper that was published not too long ago (Portoso et al., 2017), which states that HOTAIR-mediated transcriptional suppression in breast cancer cells does not always need PRC2. As a predictive biomarker, HOTAIR has the potential to be utilized in a variety of cancer types. As an illustration, it was revealed that breast cancer patients exhibit high levels of HOTAIR. that has spread to other parts of the body (Gupta et al., 2010). Both the expression of genes and the invasiveness of cancer are controlled by HOTAIR, which is dependent on PRC2-mediated histone methylation. When it comes to prostate cancer, the expression of HOTAIR is strongly expressed in CRPC, while treatments with androgens suppress its expression. Blocking HOTAIR leads to a reduction in the proliferation and invasion of CRPC cells. The mechanism of action of HOTAIR involves direct interaction with AR, which serves to shield AR from the degradation of proteins This is carried out by MDM2, an E3 ubiquitin ligase. Therefore, the overexpression of HOTAIR causes an upregulation of AR target genes in a manner that is independent of androgens. This is one of the ways where HOTAIR could potentially aid in the development of castration-resistant diseases. (Zhang et al., 2015). Suppressor of cytokine signaling 2-antisense transcript 1, or SOCS2-AS1, is an antisense transcript of SOCS2. was shown to be activated by treatment with anandrogens and overexpressed in CRPC cell lines, according to the findings of a high-throughput sequencing analysis that we conducted. Additionally, it was demonstrated that SOCS2-AS1 facilitated CRPC model cell migration and proliferation. Androgen signaling is enhanced when SOCS2-AS1 binds to AR, which in turn enhances AR-mediated epigenetic control of genes like TNFSF10, which are involved in apoptosis. (Misawa et al., 2016). This is accomplished by androgen signaling being activated. It was observed by Cui et al. that the expression of long noncoding RNA 1 (PlncRNA-1) was increased in prostate cancer. Furthermore, it was shown that disrupting the AR signaling pathway and killing cancer cells are both outcomes of lncRNA knockdown. (Cui et al., 2013). An additional research conducted not too long ago shown that PlncRNA-1 has a role in facilitating cell migration and invasion by enhancing the release of TGF-β1 (Jin et al., 2017).

A few examples of RNA-binding proteins are PSF, NONO, and paraspeckle component 1 (PSPC1). are involved in the formation of the paraspeckle structure in nuclear foci by nuclear-enriched abundant transcript 1 (NEAT1), which then controls transcription by sequestering these proteins (Hirose et al., 2014). When it comes to a number of different kinds of cancer, NEAT1 is frequently increased, and the levels of expression are related to the illness’s severity (Yu et al., 2017). NEAT1 has been shown to rank among the ERα-regulated long noncoding RNAs that are most highly overexpressed in prostate cancer., according to an integrated study of ERα occupancy and signature in prostate cancer (Chakravarty et al., 2014). A greater expression of this long noncoding RNA (lncRNA) in prostate cancer contributes to the development of resilience in the face of AR inhibitors or androgen deprivation. Based on these findings, it appears that the combination of targeting ERαand NEAT1might potentially offer a revolutionary treatment approach for individuals who are afflicted in patients with advanced breast cancer. A transcript known as TRPM2-AS is antisense. that has been identified anywhere within the TRPM2 gene, which is a subfamily M cation channel. It has been shown to be increased with melanoma (Orfanelli et al., 2008) and prostate cancer (Lavorgna et al., 2015), and the expression level is connected with a bad clinical result. It has been demonstrated through knockdown experiments that TRPM2-AS is linked to both the growth of prostate cancer cells and the death of apoptotic cells (Orfanelli et al., 2015), but the specific biochemical mechanism underlying this association is not yet fully understood.

### 4.2 LncRNAs in prostate cancer drug resistance

The most significant challenge facing cancer treatment is known as MDR. Metastatic cancer cells have the ability to evade the effects of chemotherapeutics, which can be innate or acquired (Haghighi et al., 2023). This ability is referred to as chemoresistant cells (Alfarouk et al., 2015). The development of inherent drug resistance happens when cancer cells, following the administration of chemotherapeutic medicines, raise the expression level of tumor-promoting genes while decrease the expression level of tumor-suppressor genes. This results in an increase when it comes to cell division and proliferation, along with an inhibition of apoptosis. Genetic instability and evolutionary factors were also responsible for the acquisition of drug resistance in these organisms. Generally speaking, the channels for bypass signaling, drug efflux pumps, linkages, and epigenetic changes that exist in the tumor area have the potential to result in the establishment of chemoresistance (Zhong et al., 2021). According to the findings of the research, lncRNA plays a role in the development of chemoresistance in a variety of malignancies, particularly prostate cancer. Because of this, the influence of lncRNA on drug resistance might vary depending on the function of lncRNAs and the targets they target (Ding et al., 2021). The lncRNA HOXD-AS1 is one of the lncRNAs that are implicated in treatment resistance. It is shown to be increased in CRPC cells and has a strong correlation with lymph node metastases and life without progression. The downregulation of HOXD-AS1 reduced the growth of CRPC cells as well as the development of drug resistance in both *in vitro* and *in vivo* settings. Additionally, Some genes have been linked to the cell cycle, resistance to drugs, and castration resistance have been identified and stimulated transcriptionally through the use of HOXD-AS1. These genes include UBE2C, FOXM1,CDC25C, AURKA, and PLK1, among others; Aurora kinase A is also involved. It has been established that HOXD-AS1 utilized WDR5 in order to directly modify the expression of the target genes’ expression. Overall, the recruitment of WDR5 by HOXD-AS1 is responsible for the promotion of cell division, resistance to chemotherapy, and resistance to castration in papillary carcinoma (Gu et al., 2017). A different research found that the long noncoding RNAs EGFR and LOXL1-AS1were expressed at a low level, but the doxorubicin-resistant prostate cancer DU-145 cells exhibited an overexpression of the microRNA miR-let-7a-5p. This microRNA has the potential to target the epidermal growth factor receptor (EGFR) as well as the long noncoding RNA LOXL1-AS1, which might have an impact on the course of prostate cancer. In general, The doxorubicin-resistant DU-145 cells’ migration, apoptosis, and proliferation were all profoundly affected by the lncRNALOXL1-AS1/miR-let-7a-5p/EGFR axis. which may indicate a viable therapeutic strategy for patients with drug-resistant prostate cancer (Bai et al., 2019).

In docetaxel-resistant prostate cancer samples, NEAT1 was found to be overexpressed, as was indicated before. NEAT1 was silenced, which led to a reduction in the amount of cell proliferation and invasion that occurred in PCa cells that were resistant to docetaxel. Through the act of miR-34a-5p and miR-204-5p sponging in prostate cancer cells, NEAT1 plays a functional role in the development of docetaxel resistance (Jiang et al., 2020). This is accomplished by increasing the expression of ACSL4. The expressions of another long noncoding RNA, CCAT1, were demonstrated to be overexpressed in PCa cells that were resistant to either paclitaxel or PTX. Following treatment with PTX, the suppression of CCAT1 led to a reduction in the survival rate of cells and an increase in the rate of apoptosis (Li X. et al., 2020). The expression of the long noncoding RNA SNHG6 was also shown to be increased in drug-resistant prostate cancer tissues and cells. Experimentally and clinically, the suppression of SNHG6 led to an increase in the susceptibility of PTX-resistant prostate cancer cells to the drug. Additionally, the suppression of SNHG6 reduced PTX-resistant PCa cell migration, invasion, and proliferation *in vitro*. It has been suggested that SNHG6 may have the potential to be a therapeutic factor for prostate cancer (Cao C. et al., 2020). This is because reducing SNHG6 levels made PTX-resistant PCa cells more vulnerable. to PTX by acting as a tumor suppressor against miR-186. There was also an increase in the expression of Linc00518 in PCa, which was associated with paclitaxel resistance. The lack of Linc00518 in PCa cell lines resulted in a reduction in their resistance to PTX (He et al., 2019). In PCa that was resistant to docetaxel or DTX, DANCR was shown to be highly elevated. Suppressing DANCR caused a rise in the effectiveness of DTX in PCa cells that were resistant to DTX (Ma et al., 2019).

The activation of alternative routes for AR signaling renders PC cells insensitive to ADT, leading to this outcome. which is a fundamental stance against. Castration-resistant prostate cancer (CRPC) is considered a more advanced type of cancer that coincides with the fact that patients have a low survival rate. LncRNA is responsible for controlling several of these routes. Xenograft tissues derived from patients with neuroendocrine prostate cancer (NEPC) who have developed a resistance to hormonal therapies show an overexpression of lncRNA-p21., according to a research that is rather intriguing. Additionally, it has been demonstrated that the antiandrogen enzalutamide (Enz), which is a medicine that is successful in increasing the survival rate of patients with CRPC, also enhances the expression of lncRNA-p21, as a result of which neuroendocrine differentiation (NED) occurs. In addition, functional *in vitro* investigation demonstrated that cell exposure to Enz resulted in the overexpression of lncRNA-p21 through the modulation of AR activity. This, in turn, led to the activation of STAT3 signaling through the Enhancer of zeste homolog 2 (EZH2) pathway. Several studies have shown that this particular signaling pathway plays a role in the process of fostering neuroendocrine differentiation. In addition, research that took place in living organisms revealed that inhibiting In mouse models, EZH2 was able to mitigate the neuroendocrine differentiation generated by Enz therapy. This finding suggests that targeting lncRNA-p21 could be an effective strategy for better management of patients with colorectal cancer who are battling the progression of non-epithelial squamous cell carcinoma (Luo et al., 2019). An further carcinogenic long noncoding RNA (lncRNA) that plays a role in the development of CRPC is called LncRNA-PCAT1. PTEN-deficient individuals experience castration resistance as a result of the activation of the AKT signaling pathway, which is caused by the inhibition of AR signaling signals. There is a report that LncRNA-PCAT1 has the capacity to interfere with a crucial regulatory complex that comprises an inhibitor of nuclear factor kappa B (IKKα) FKBP51,PHLPP, and PH domain. This disruption occurs through the interaction of LncRNA-PCAT1 with FKBP51, which results in the displacement of PHLPP from the complex. This, in turn, activates the signaling pathways of AKT and Nuclear factor kappa B (NF-κB).

### 4.3 LncRNAs as biomarkers in prostate cancer

PCA3, which was initially found in 1999 using prostate tissue and cell line differential display analysis, is considered to be one of the most precise biomarkers for prostate cancer (Bussemakers et al., 1999). While its expression was found to be sixty to one hundred times greater in more than ninety-five percent of prostate cancers in comparison to non-neoplastic tissues that were adjacent to the tumors, it was not detected in any other forms of malignancies. The fact that knocking down PCA3 reduces AR signaling, as well as cell growth and survival, suggests that modulating AR signaling in tumor cells may be possible by overexpression of PCA3. There is a partial elevation of epithelial indicators such as cytokeratin-18, claudin-3, and E-cadherin when PCA3 is knocked down, while there is a downregulation of the mesenchymal marker vimentin (Lemos et al., 2016). Additionally, PCA3 is responsible for regulating the expression of significant genes that are associated with cancer and are associated with mitogen-activated kinase 1, cell adhesion, signal transduction, apoptosis, and angiogenesis. (Lemos et al., 2016). Further, a PCA3 operational model is now under consideration. According to this model, PCA3 functions as a dominant-negative oncogene that suppresses the activity of the unidentified tumor suppressor Prune Homolog 2 (PRUNE2)), which is the prune gene in fruit fly hybrids with its human equivalent. The procedure relies on RNA editing, namely, the production of double-stranded RNA, to achieve this goal. that is PRUNE2/PCA3 (Salameh et al., 2015). When compared with serum PSA, the combination of urine PCA3 and fusion gene TMPRSS2-ERG has the potential to significantly reduce the number of prostate biopsies that are not necessary. This combination can also boost the specificity of the diagnosis of prostate cancer. The long noncoding RNA known as SChLAP1, which stands for second chromosomal locus associated with prostate is significantly expressed in twenty-five percent of prostate cancer cases (Prensner et al., 2013). There is a substantial correlation between its expression and the likelihood of mortality, clinical progression, biochemical recurrence, metastasis specifically related to prostate cancer. In cases of colorectal cancer, its expression is higher. By interacting with the Switch-Sucrose Non-Fermentable (SWI/SNF) complex for the purpose of chromatin remodeling, SChLAP1 is able to reverse the effects of SWI/SNF, which are known to decrease tumor growth (Prensner et al., 2013). Biochemical recurrence after radical prostatectomy can be independently predicted by this lncRNA., according to an analysis of SChLAP1 expression using *in situ* hybridization (ISH) (Mehra et al., 2014). This long non-coding RNA (lncRNA) is a useful biomarker for prostate cancer patients that is found in tissues. who are at a greater risk of CRPC advancement. Furthermore, the expression of SChLAP1 was found to connect with the progression of prostate cancer that was likely to be fatal (Mehra et al., 2016). In normal prostate tissues and non-cancerous prostate epithelial cells, the expression of the long noncoding RNA known as SPRY4 intronic transcript 1 (SPRY4-IT1) is seen to be much higher in patient samples and inPC3 cells (Lee et al., 2014). siRNA knockdown of SPRY4-IT1 decreased the spread of PC3 cells and their invasion, and also increased the number of cells that underwent apoptosis. According to the results of an RNA chromogenic ISH test, SPRY4-IT1 was easily identified in all prostate cancer samples with varying Gleason scores ranging from 6 to 10 (Lee et al., 2014). Due to its selectivity for prostate cancer and its ability to be easily detected using conventional clinical staining methods on tissue samples, this long noncoding RNA is a promising candidate for use as a diagnostic biomarker. MALAT1, which stands for metastasis-associated lung adenocarcinoma transcript 1, is a long noncoding RNA that was initially discovered to may be overexpressed in tissues of non-small-cell lung cancer patients with a high propensity to metastasize? (Ji et al., 2003). Recent research has demonstrated that MALAT1 is also overexpressed in various types of human cancer, such as those that affect the breast, pancreatic, colon, prostate, and liver (Lin et al., 2007; Konishi et al., 2016). MALAT1 overexpression was shown to be related with markers of poor prognosis in prostate cancer, which includes a high Gleason result, advanced stage of tumor node metastasis, and serum PSA levels that were greater than 20 ng/mL. Furthermore, the expression of MALAT1 was considerably higher in hormone-resistant prostate cancer (CRPC) than in cases of prostate cancer that detect hormones (Ren et al., 2013). A study that analyzed MALAT1 expression in prostate cancer patients whose biopsies came back positive and those whose did not found the disease, this lncRNA was shown to be considerably greater in biopsy-positive samples (Wang et al., 2014). This finding suggests as a potential diagnostic biomarker, urine MALAT1 could be useful. By combining EZH2-antibody RNA immunoprecipitation with high-throughput sequencing analysis, it was also determined that MALAT1 binds to EZH2. (Wang et al., 2015). A favorable link between MALAT1 and EZH2 has been shown, and it has been suggested that MALAT1 plays a significant part during the course of CRPC cell line migration and invasion facilitated by EZH2 (Wang et al., 2015; Misawa et al., 2017). Therefore, increasing evidences demonstrate that lncRNAs are potential regulators of tumorigenesis in prostate cancer (Zhang A. et al., 2016; Ramnarine et al., 2019; Ma G. et al., 2016).

### 4.4 LncRNAs in autophagy regulation in prostate cancer

**FIGURE 3:**
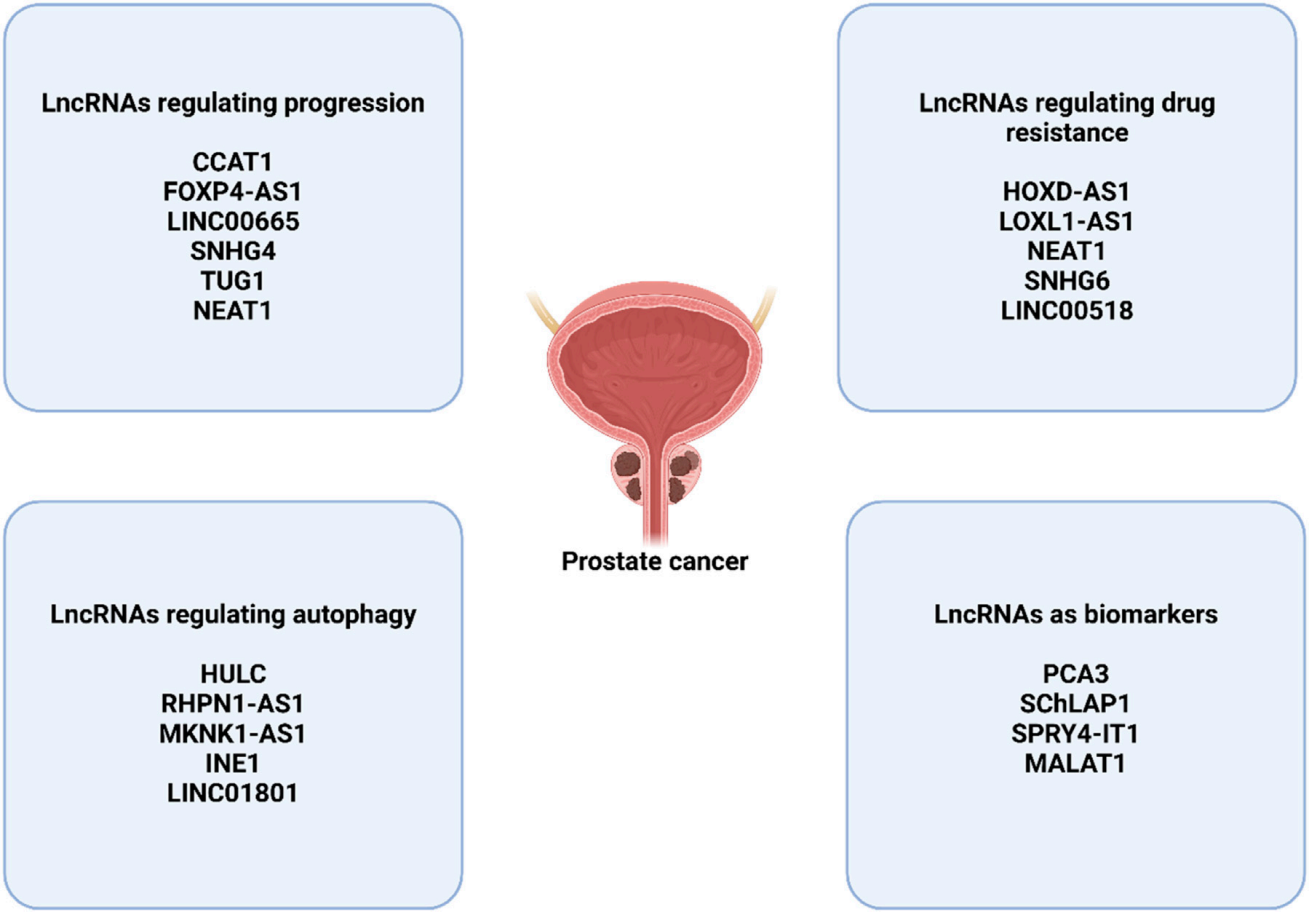
The function of lncRNAs in prostate cancer.

A few of studies have evaluated the function of lncRNAs in the regulation of autophagy in prostate cancer. The high expression of lncRNA HULC can promote the survival. The HULC silencing can reduce survival rate and enhance apoptosis in prostate cancer. HULC downregulation increases radiosensitivity and stimulates autophagy through Beclin-1 upregulation and mTOR downregulation (Lambert et al., 2018). The lncRNA RHPN1-AS1 downregulation can stimulate apoptosis and autophagy in prostate cancer. LncRNA RHPN1-AS1 sponges miR-7-5p to upregulate EGFR for induction of mTOR to suppress autophagy (Ma X. et al., 2022). On the other hand, the function of REST in the suppression of LINC01801 can transcriptionally stimulate autophagy in enhancing neuroendocrine differentiation of prostate cancer (Chang et al., 2023). Moreover, MKNK1-AS1 and INE1 have been identified as autophagy-related lncRNAs that determine the survival rate of prostate cancer (Li et al., 2021). Figure 3 highlights the function of lncRNAs in prostate cancer.

## 5 LncRNAs and bladder cancer

### 5.1 LncRNAs in bladder cancer progression

Different kinds of lncRNAs can be distinguished from one another on the basis of their function, genomic location, and subcellular localization (Cao Y. et al., 2020). There are five different types of lncRNAs that are categorized according to their position in the genome. The long non-coding RNAs can be grouped into several types, such as sense, antisense, bidirectional, intergenic, and intronic. One example of an intergenic long noncoding RNA is H19, another is UCA1, and a third is MALAT1. (Ariel et al., 2000; Xue et al., 2017; Jiao et al., 2018). Intronic lncRNAs includelncRNA-LET, SPRY4-IT1, and BLACAT1 (He et al., 2013; Zhao et al., 2015; Zhuang et al., 2017a). Antisense long noncoding RNAs (lncRNAs) include SNHG16 and GAS5 (Cao et al., 2018; Avgeris et al., 2018), GAS5 transcripts can be either coding RNA or bidirectionally long noncoding RNA. (Wang et al., 2018). On top of that, Two groups are composed of long noncoding RNAs.: nuclear lncRNAs and cytoplasmic lncRNAs, depending on where they are located inside the cell. BLACAT2 and LBCS were shown to be localized in the nucleus of bladder cancer cells, according to the results of investigations involving subcellular fractionation and *in situ* hybridization (ISH) (He W. et al., 2018; Liu P. et al., 2019). Both ARAP1-AS1 and LSINCT5 were shown to be abundant in the cytoplasm of BC cells, as opposed to other proteins (Zhu et al., 2018; Teng et al., 2019). In addition, long noncoding RNAs are categorized into four categories based on the roles that they perform: guide, decoy, signaling, and scaffold lncRNAs. As an illustration, LNMAT1 was responsible for the promotion of lymphatic metastasis of bladder cancer. This was accomplished via enhancing CCL2 promoter recruitment of hnRNPL, which increased the production of CCL2 (Chen et al., 2018). DBCCR1-003 has the potential to bind to DNMT1 and so block the methylation of DBCCR1 in BC that is mediated by DNMT1. Following this, the overexpression of DBCCR1-003 resulted in a considerable reduction in the proliferation of bladder cancer cells as well as the death of these cells (Zhuang J. et al., 2015). Through the process of sponging miR-101-3p, SPRY4-IT1 was able to increase the rate of bladder cancer cell growth and spread (Liu D. et al., 2017). This was accomplished by upregulating zeste homologue 2 (EZH2). In addition, long noncoding RNAs (lncRNAs) are capable of performing their tasks within the transcriptional levels, after the fact, and regulation of epigenetics, independent of the categories that they fall under. The long noncoding RNA (lncRNA) is a regulatory gene that has the potential to exert a significant effect on several biological processes. These activities include cell death, cell proliferation, cell maturation, and cell specialization. For example, Luo et al. reported that an increase in the expression of H19 led to an increase in the proliferation of bladder cancer cells (Luo et al., 2013). When compared with normal tissues, the prevalence of GAPLINC was shown to be considerably higher in bladder cancer tissues. The inhibition of GAPLINC led to the promotion of cell cycle arrest at the G1 phase, as well as the inhibition of a capacity to migrate and invade (Zheng et al., 2018). A similar effect was observed when SNHG16 was knocked down, which led to the halt of the cell cycle at the G1 phase and enhanced apoptosis in bladder cancer cells (Cao et al., 2018). Through its interaction with WDR5, overexpressed BLACAT2 was able to generate intratumoral and peritumoral lymphangiogenesis, which in turn increased the invasiveness of bladder cancer cells (He W. et al., 2018). Furthermore, Not only did MEG3 overexpression inhibit cell invasion and migration, but it also made bladder cancer cells more responsive to cisplatin, a chemotherapeutic agent. (Kim and Tannock, 2005).

### 5.2 LncRNAs in bladder cancer therapy resistance

In clinical practice, chemotherapy is the first-line treatment for breast cancer, and it is effective in reducing tumor masses in the majority of patients (Zhang et al., 2021). However, after repeated treatment cycles, the majority of patients gradually lose their ability to respond to treatment, and they eventually experience a recurrence of their tumor (Kurtova et al., 2015). The chemotherapeutic response in BCa has been demonstrated to be altered by a number of different long noncoding RNAs. Cisplatin, a fundamental substance used in the initial phase of chemotherapy treatment, has been demonstrated to dramatically enhance the prognosis in patients who are sensitive to the treatment (Herr et al., 2007). Through its role as an oncogene, TUG1 is able to directly sponge miR-194-5p and promote the production of EZH2. There is a correlation when miR-194-5p levels are low and CCND2 expression is high which causes BCa cells to become more resistant to the chemotherapy drug cisplatin (Yu et al., 2019). In addition to this, increasing the sensitivity of BCa cells to adriamycin is achieved by TUG1 knockdown (Sun Z. et al., 2019). A knockdown of LINC00857 makes breast cancer cells more sensitive to cisplatin. This is accomplished via controlling the expression of the LMAN1 gene, which suggests that LINC00857 has the ability to modulate sensitive patient responses to platinum-based chemotherapy (Dudek et al., 2018). A high level of HIF1A-AS2 in cisplatin-resistant breast cancer cells causes an increase in the production of HMGA1, which in turn limits the transcriptional activity of proteins belonging to the p53 family. This, in turn, has an effect on the apoptosis that is caused by cisplatin (Shin et al., 2019). According to the findings of a recent study (Li Y. et al., 2019), When DLEU1 restores the expression of the target gene HS3ST3B1, it improves cisplatin resistance through competitive regulation of miR-99b. It has been demonstrated that the downregulation of MALAT1 increases the susceptibility of BCa cells to cisplatin through the miR-101-3p/VEGFC axis (Liu P. et al., 2019). The susceptibility of breast cancer cells to cisplatin has been discovered to be suppressed by MST1P2, which regulates miR-133b/SIRT1 signaling (Chen J. et al., 2020). It has been demonstrated that UCA1 can reduce the susceptibility of BCa cells to cisplatin by increasing the expression of Wnt6 (Fan et al., 2014a). In addition, long noncoding RNAs have the ability to boost the chemosensitivity of breast cancer cells to cisplatin and suppress treatment resistance. As an illustration, the overexpression of MEG3 may cause BCa cells to become more sensitive to the chemotherapeutic medication cisplatin (Feng et al., 2018).

Another cytotoxic chemotherapeutic drug that is used to treat BCa cells is gemcitabine; nevertheless, the majority of patients, in a manner comparable to those who were treated with cisplatin, ultimately experience a recurrence of their tumors (Kim and Tannock, 2005). When gemcitabine is used as a treatment, the increase of LET makes it more difficult for BCa to return. It is worth noting that the proinflammatory cytokine TGFβ1 has the ability to directly reduce the levels of LET expression in individuals who are resistant to gemcitabine (Zhuang et al., 2017b). However, FOXD2-AS1 is responsible for the positive regulation of ABCC3 protein through the targeting of miR-143. Evidence suggests that this protein’s knockdown suppresses not only the 50% inhibitory concentration of gemcitabine but also invasion, the expression of ABCC3 protein in gemcitabine-resistant BCa cells, and drug resistance-related genes (MDR1, LRP1 MRP2). (An et al., 2018). There is a correlation between high levels of CDKN2B-AS expression and poor gemcitabine sensitivity. Conversely, the Wnt signaling pathway is rendered inactive by decreased levels of the CDKN2B-AS gene, which eventually has an effect on the sensitivity of BCa cells to gemcitabine (Xie et al., 2018). There is a correlation between the high expression of GHET1 and the poor gemcitabine sensitivity in patients with breast cancer, and the knockdown of GHET1 is related with an increase in gemcitabine-induced cytotoxicity (Li B. et al., 2019). In addition, UCA1 is responsible for the activation of the transcription factor CREB by its interaction with its promoter, which ultimately results in the production of miR-196a-5p. Conversely, the inhibition of UCA1 leads to a reduction in chemosensitivity to cisplatin and gemcitabine by reducing the proliferation of BCa cells (Pan et al., 2016). It has been discovered via additional research that lncRNAs also have a significant role in the chemosensitivity of BCa to doxorubicin. Doxorubicin induces cell death, and an increase in GAS5 decreases treatment resistance to doxorubicin. (Shang et al., 2016; Zhang et al., 2017). Increased cell proliferation and decreased doxorubicin chemosensitivity are effects of HOTAIR overexpression., whereas doxorubicin induces cell death. TUG1 role in EMT and radioresistance is mediated via the miR-145/ZEB2 axis, which is responsible for the radioresistance of BCa. Reduced expression of TUG1 enhances radiosensitivity in BCa by repressing the targeting gene The HMGB1 gene (Jiang et al., 2017a; Jiang et al., 2017b).

### 5.3 LncRNAs as biomarkers in bladder cancer

The expression of thirteen potential long noncoding RNAs was recently assessed by Duan et al. in bladder cancer that was matched to healthy tissue in the surrounding area. They reported a panel of lncRNAs that were expressed differently, and these lncRNAs were then examined using blood samples. There was a discernible difference in the expression of three long noncoding RNAs (MALAT1, SNHG16, and MEG3) in the blood of healthy persons in contrast to serum from both cancerous and noncancerous bladder diseases (Taheri et al., 2018; Duan et al., 2016). It is possible that this panel could aid patients in detecting bladder cancer. There is a statistical correlation between the histological grade and TNM stage of bladder cancer and the expression of several lncRNAs in this malignancy. (Zhuang C. et al., 2015; Zhan et al., 2016a; Zhan et al., 2016b; Chen M. et al., 2016; Li J. et al., 2016; XianGuo et al., 2016). These lncRNAs include HIF1A-AS2, SUMO1P3, PANDAR, CCAT2, PVT1, and NEAT1. Furthermore, according to Chen et al. (Chen et al., 2015), there is a positive correlation between the expression of lncRNA-n336928 and the stage of the bladder tumor, the histological grade, and the patient’s survival. There is a correlation between GHET1 overexpression and tumor growth, low survival rates, lymph node status, and the existence of advanced lymph nodes (Li et al., 2014). In bladder cancer, GHET1 expression is more than in surrounding tissues that are unaffected. The presence of lymph node metastases in these individuals is linked to elevated levels of MALAT1 expression, which is also connected with higher grades of histological evaluation and the stage of the tumor (Li et al., 2017). According to other studies (Li et al., 2017; Fan et al., 2014b), the presence of MALAT1 overexpression is a leading indicator of poor survival in these individuals. There is a correlation among patients with muscle-invasive bladder cancer and elevated TUG1 levels in their metastatic tumors (Iliev et al., 2016). TINCR expression levels, on the other hand, have just been established as being related with advanced TNM stage (Chen et al., 2016b). In contrast, a positive correlation was found between low expression of BANCR and MIR31HG and the TNM stage (He et al., 2016a; He et al., 2016b). Moreover, a decrease in the expression of MEG3 is linked to a decrease in the percentage of patients who survive without recurrence (Duan et al., 2016). In bladder cancer, lower GAS5 levels are linked to higher pathological grades and a lower disease-free survival rate. (Zhang et al., 2017).

### 5.4 LncRNA/ceRNA axis in bladder cancer

Cancer cell stemness, a characteristic of cancer cells that is similar to that of stem cells, has been demonstrated to have a significant role in the development of tumors, the processes of metastasis and recurrence, as well as the development of treatment resistance (Li K. et al., 2023; Tsui and Chan, 2020; Lee et al., 2022). When it comes to human malignancies, particularly bladder cancer, it has been established that lncRNA-mediated ceRNA networks play a role in the creation and maintenance of cancer cell stemness. Zhan et al. (Zhan et al., 2020) discovered bladder cancer was associated with elevated expression of the sex-determining region Y-box2 (SOX2) overlapping transcript (SOX2OT). Furthermore, they found that bladder cancer stem cells were more likely to undergo self-renewal, migration, invasion, and tumorigenicity when SOX2OT expression was up. This was accomplished by means of miR-200c “sponging” and, as a result, increasing SOX2 expression, which is an essential regulator of cancer stemness (Zhu et al., 2021; Mamun et al., 2020). Furthermore, it was shown that through its modulation of the miR-125b/smad2 axis, the oncogenic long noncoding RNA HOXA cluster antisense RNA 2 (HOXA-AS2) enhances the stemness of bladder cancer cells by elevating the expression levels of cancer stem cell markers like OCT4. KLF4, CD44, HMGA2, and ALDH1A1, (Wang F. et al., 2019). Furthermore, it has been reported that a specific type of antisense RNA known as potassium calcium-activated channel subfamily M regulation beta subunit 2 (KCNMB2-AS1) has the ability to improve the stemness of bladder cancer cells. This is accomplished via modulating the miR-3194-3p/smad5 signaling pathway, which in turn increases the expression of cancer stem cell markers like ALDH1, Oct4, Nanog, CD133, and Nanog. (Chen et al., 2021). Microfilaments, microtubules, and intermediate filaments are the components that make up the eukaryotic cytoskeleton, which is distinguished by its intricate fibrous reticular structure. A growing body of data has proven the cytoskeleton is responsible for signal transduction, cell motility, intercellular transport, and cell division. As a consequence, the cytoskeleton plays a part in the uncontrolled proliferation and migration of cells that occur throughout the evolution of cancer (Eli et al., 2022; Datta et al., 2021). It has been revealed that the lncRNA-mediated ceRNA network is responsible for the rearrangement of the cytoskeleton in the advancement of bladder cancer. For example, Lv et al. (Lv et al., 2017) discovered both human bladder cancer tissues and cell lines exhibit elevated levels of lncRNA H19. Furthermore, they discovered that cytoskeleton reorganization results from overexpression of lncRNA H19. This is accomplished via boosting paxillin and F-actin expression, which are a pair of cytoskeletal proteins involved in cancer cell movement, adhesion, signal transduction, and motor activity (Kim et al., 2009).

Surgical procedures, chemotherapy, and radiation therapy are the conventional therapies for bladder cancer now available. On the other hand, there is a subset of individuals who have bladder cancer who remain refractory to chemotherapy or radiation, and as a result, they have a recurrence of their tumor (Patel et al., 2020; Hensley et al., 2022). In order to achieve improved outcomes for patients with bladder cancer, one of the most significant challenges is to overcome resistance to chemotherapy and radiation. Multiple studies have found that lncRNAs are associated with the ceRNA network and the development of radiation or chemotherapy resistance in bladder cancer. Based on these findings, they discovered networks that target lncRNA-mediated ceRNA might potentially make cancer cells more sensitive to doxorubicin, gemcitabine, and cisplatin. Additionally, along the miR-145/ZEB2 pathway, the lncRNA TUG1, which is significantly expressed at an elevated level in bladder cancer samples and cells, promotes epithelial-mesenchymal transition (EMT) and reduces the susceptibility of cancer cells to ionizing radiation (Tan et al., 2015). By suppressing the production of HMGB1, the promotion of metastasis by a conserved nuclear protein in a variety of malignancies, TUG1 silencing was shown to improve radiosensitivity in a xenograft model, according to the findings of another study (Jiang et al., 2017b; Tripathi et al., 2019). Furthermore, Recent studies that looked at lncRNA signatures in bladder cancer patients who had radiation therapy found that molecular mechanisms related to radiation responses are connected with a 10-lncRNA signature. Furthermore, A small rise in radiosensitivity was observed in bladder cancer cells when one of these lncRNAs was knocked down. (Khan et al., 2021).

### 5.5 LncRNAs in autophagy regulation in bladder cancer

**FIGURE 4:**
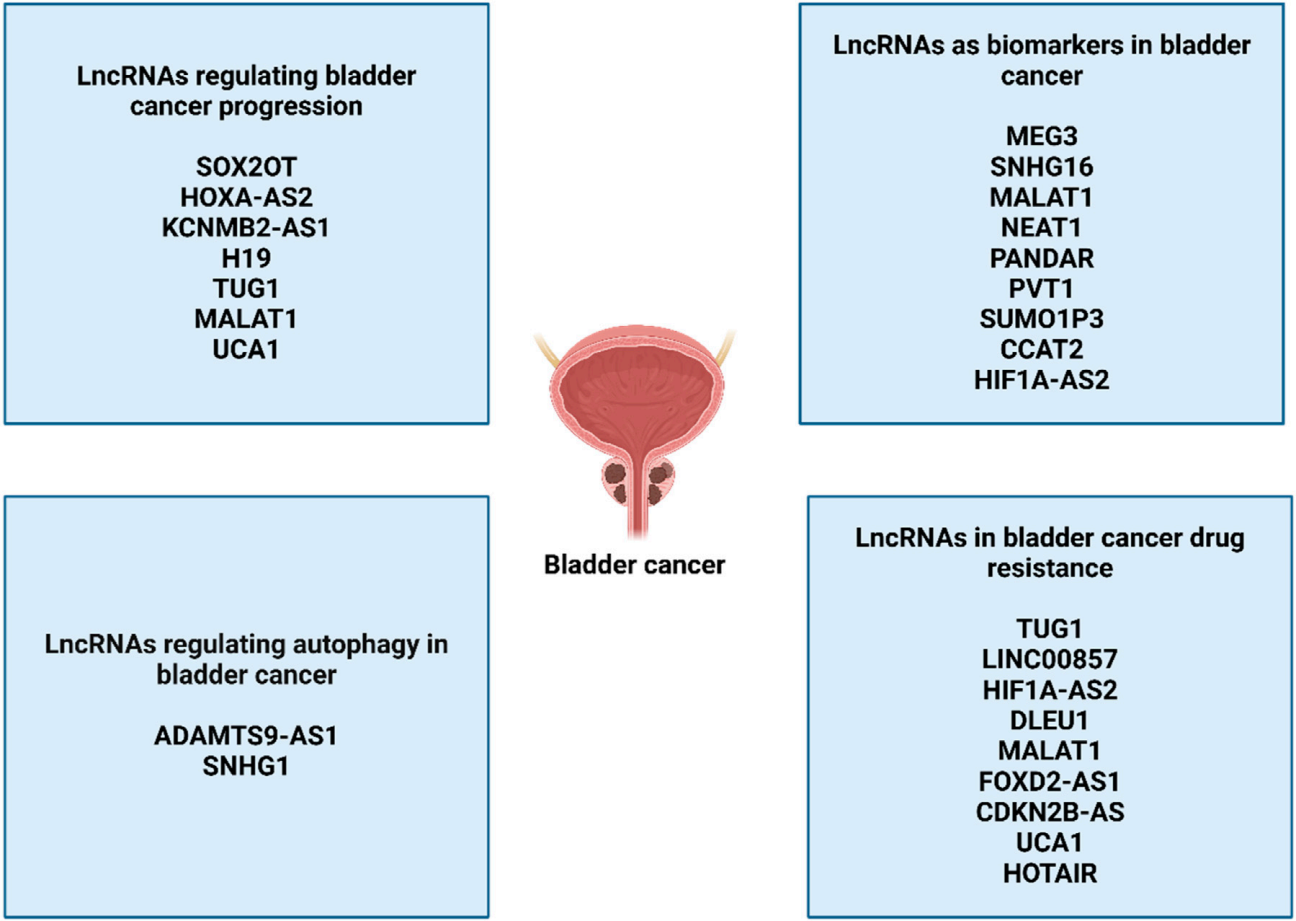
The function of lncRNAs in bladder cancer.

The lncRNAs are also potential regulators of autophagy in bladder cancer. The lncRNA SNHG1 is able to interact with catalytic subunit PP2A and stimulate autophagy to enhance metastasis of bladder cancer (Xu et al., 2020). The lncRNA ADAMTS9-AS1 stimulates PI3K/Akt/mTOR axis to suppress apoptosis and autophagy in bladder cancer (Yang et al., 2021). In spite of these discussions, more efforts are required regarding understanding the role of lncRNA-mediated autophagy regulation in bladder cancer (Figure 4).

## 6 LncRNAs and renal cancer

### 6.1 LncRNAs in renal cancer progression and drug resistance

The lncRNAs have been considered as regulators of drug resistance in kidney cancer (Barth et al., 2020). The sorafenib resistance-associated long noncoding RNA (SRLR) in RCC was firstly tested for functionality by Xu and colleagues. (Xu et al., 2017), who mentioned that SRLR was shown to improve treatment resistance with sorafenib, a multi-kinase inhibitor. Tissue from sorafenib-resistant patients and cells from skin cancer patients both showed an upregulation of SRLR. In terms of the mechanism, SRLR has a direct interaction with the transcription factor NF-KB, which then leads to the stimulation of IL-6 transcription and release of IL-6 by RCC cells in an autocrine manner. The inhibition of receptor tyrosine kinases, such as VEGFR and PDGFR, by sorbafenib is circumvented as a consequence of this, which leads to the activation of the STAT3 pathway. It was demonstrated through experiments that this is true both *in vitro* and *in vivo* (Xu et al., 2017). In a research that looked at SRLR in polycystic ovarian syndrome (Saab et al., 2020), the link between SLRL and IL-6 was only recently verified because it was only just discovered. Higher expression levels of the long noncoding RNA SRLR were found to be associated with a decreased progression-free survival (PFS) in a clinical dataset consisting of 95 patients with recurrent colorectal cancer (RCC) (hazard ratio = 0.407, 95% confidence interval = 0.222–0.744, *p* = 0.003). Furthermore, this association was also associated with low levels of IL-6 and a lack of benefit from sorafenib treatment. A major influence on resistance to treatment with sunitinib, a multikinase inhibitor, for RCC is exerted by the long noncoding RNA (lncRNA) that is activated in RCC patients who have sunitinib resistance (ARSR) (Qu et al., 2016). Through a mechanism that involves functioning as a competitive endogenous RNA (ceRNA), ARSR is able to sequester miR-34 and miR-449, which in turn leads to a rise in the concentrations of the endpoints AXL and c-MET, which ultimately leads to the promotion of sunitinib resistance. Through sunitinib-resistant cell lines, the ARSR gene is overexpressed, and in turn, By activating FOXO transcriptional factors, AXL enhances the expression of the ARSR gene. This indicates that there is a positive feedback loop between AXL and ARSR in kidney cancer that is resistant to sunitinib. The transfer of sunitinib resistance from cells that are resistant to sunitinib to cells that are sensitive to sunitinib can also occur through the process of exosome-mediated transmission, which is an intriguing phenomenon. *In vivo* and *in vitro* research have demonstrated that targeting ARSR could be considered as a possible treatment option for sunitinib resistance. (Qu et al., 2016). Both of these experiments were conducted. These findings are supported by the fact that pretreatment ARSR levels in the plasma of RCC patients are substantially connected with poor progression-free survival (PFS) for high vs. low ARSR expression (hazard ratio = 2.9, 95% confidence interval = 1.2–7.1, *p* = 0.017), respectively (Qu et al., 2016). The ARSR sequence’s single nucleotide polymorphisms were also recommended as possible biomarkers for the outcome of RCC in a research that was conducted not too long ago. Numerous investigations have demonstrated which NEAT1—the nuclear paraspeckle assembly transcript and its role as an oncogenic long noncoding RNA have already been thoroughly examined (Klec et al., 2019). There is evidence that NEAT1 contributes to the development of resistance to chemotherapy (Shin et al., 2019; An et al., 2017). Because it acts as a sponge for miR-34a, NEAT1 may be able to block the response to sorafenib therapy in RRC. This is accomplished through the control of the NEAT1/miR-34a/c-MET axis (Liu F. et al., 2017). There have been previous reports that c-MET and miR-34a have an effect on chemoresistance in various types of cancer, such as osteosarcoma and esophageal cancer (Hara et al., 2019; Sun Z-Y. et al., 2019; Pu et al., 2017). Furthermore, NEAT1 has a great deal of expression in RCC cell lines as well as tissues. In addition, there was a correlation between the overexpression of NEAT1 and the change from epithelial to mesenchymal (EMT), as well as a substantial correlation with poor overall survival and progression-free survival in lung cancer. However, the study conducted by Liu et al. did not include any univariate or multivariate analyses, nor did it include any xenograft models (Liu F. et al., 2017).

It has already been established that the long noncoding RNA ADAMTS9 antisense RNA 2 (ADAMTS9-AS2) plays a role in the development of treatment resistance in cancer. Tamoxifen resistance is worsened by ADAMTS9-AS2 downregulation in breast cancer. but its downregulation was related with improved sensitivity to temozolomide in glioblastoma (Yan et al., 2019; Shi et al., 2019). This suggests that its role may vary depending on the kind of cancer being treated. Downregulation of ADAMTS9-AS2 is seen in RCC, and a substantial association is shown between high expression and improved overall survival (Song et al., 2019). Increasing the expression of FOXO1 and restoring chemosensitivity to 5-fluorouracil and cisplatin were both outcomes of overexpressing ADAMTS9-AS2, which was accomplished by the sequestration of miR-27-3p. Nevertheless, there is a lack of evidence carried out in in vivo tests (Song et al., 2019). To this day, chemotherapy is not a viable therapeutic choice for RCC since it has been demonstrated to be unsuccessful; hence, the direct practical significance of the study is restricted (Amato, 2000). Targeting long noncoding RNAs, on the other hand, has the potential to overcome chemoresistance in RCC in the future and open the door for chemotherapy to be considered a viable therapeutic choice for RCC. In a study that was conducted by Liu and colleagues (Liu L. et al., 2019), it was discovered that the long noncoding RNA known as growth arrest specific transcript 1 (GAS5) has an effect on the resistance of RCC to sorafenib. It has already been proven on several occasions (Ma C. et al., 2016) that GAS5 has a tumor suppressive function in the development and progression of reactive phase carcinoma. In terms of its influence on sorafenib resistance, it was demonstrated that it acts as a sponge for miR-21. Furthermore, it was found that the elevation of GAS5 led to the upregulation of the transcription factor sex determining region Y-box protein 5 (SOX5), which in turn conferred enhanced sensitivity to sorafenib (Ma C. et al., 2016). Multiple models, both *in vitro* and *in vivo*, were used to demonstrate this statement. These findings are supported by the fact that all of the effectors in the GAS5/miR-21/SOX5 pathway, as hypothesized by Liu et al. (Ma C. et al., 2016), have already been found to be effectors in chemoresistance on their own (Gao et al., 2018; Chen Z. et al., 2020; Chen et al., 2019; Dai et al., 2017).

### 6.2 LncRNAs as diagnostic and prognostic factors in renal cancer

There have been a number of research studies that have focused on lncRNAs with the objective of identifying new biomarkers and gaining a knowledge of the molecular processes that they use to impact the beginning and development of recurrent cardiac tumors (Outeiro-Pinho et al., 2020; Song et al., 2014; Wang et al., 2017; Xue et al., 2019). When compared to their counterparts that code for proteins, lncRNAs are far less expressed. This might be a significant obstacle for their application in clinical practice, since it is extremely difficult to identify them in a reliable manner (Mattick and Rinn, 2015). The investigation of these compounds need to be encouraged, despite the fact that technical advancements might be able to overcome the limits that are currently in place. The most pertinent research that reported lncRNAs as possible diagnostic, prognostic, predictive, and monitoring biomarkers in randomized controlled trials (RCTs) were emphasized in this article. These investigations were conducted on tissue and liquid biopsies. As opposed to sncRNAs, there is a dearth of published information about lncRNAs as diagnostic biomarkers for randomised controlled trials. More than 20 years ago, Thrash–Bingham and colleagues (Thrash-Bingham and Tartof, 1999) made the groundbreaking discovery that the expression of lncRNA varied not only between RCC subtypes but also between subtypes of RCC. It was discovered through the use of semiquantitative PCR that the expression of lncRNA antisense Hypoxia Inducible Factor (aHIF) was significantly higher in ccRCC in comparison to pRCC (Thrash-Bingham and Tartof, 1999). Technology has advanced, and these findings were subsequently verified in 2011, when Bertozzi and colleagues (Bertozzi et al., 2011) discovered a differential expression of lncRNA aHIF between RCC and MNT, as well as between non-pRCC and pRCC tissue samples. This was one of the first times that these findings were validated. In a different research, which included 102 ccRCC and 50 NRT, the lncRNA CYP4A22–2/3 was able to differentiate between ccRCC and NRT with an area under the curve (AUC) of 0.790 (Ellinger et al., 2015). Ren and his colleagues (Ren et al., 2016) conducted an investigation in 2016 to determine the level of expression of the long noncoding RNAs UC009YBY.1 and ENST00000514034 in a collection of 70 ccRCC and 70 MNT cells. These authors observed that the two lncRNAs were able to detect RCC tissue with a sensitivity of 54.29% and a specificity of 82.86% for the former, and with a sensitivity of 60.00% and a specificity of 67.14% for the latter (Ren et al., 2016). Last but not least, a recent research revealed that the lncRNA HOX Transcript Antisense RNA (HOTAIR) might potentially serve as a diagnostic biomarker for colorectal cancer, uncovering an area under the curve (AUC) of 0.9230 (Dasgupta et al., 2018). After doing a search of the relevant literature, we discovered that there were only two publications that were relevant to the evaluation of the potential of lncRNAs as RCC diagnostic biomarkers in liquid biopsies. Using two different sets of ccRCC and AC serum samples, Wu and colleagues (Wu et al., 2016) investigated the expression of five different long non-coding RNAs (lncRNAs): lncRNA–low expression in tumor (LET), Plasmacytoma Variant Translocation 1 (PVT1), Promoter of CDKN1A Antisense DNA Damage Activated RNA (PANDAR), Phosphatase and Tensin Homolog Pseudogene 1 (PTENP1), and long intergenic non-protein RNA 963 (linc00963). These biomarkers, when integrated in a panel, were able to identify malignancy with a sensitivity of 79.2% and a specificity of 88.9% in the training set (consisting of 24 ccRCC and 27 AC), and with a sensitivity of 67.6% and a specificity of 91.4% in the testing set (consisting of 37 ccRCC and 35 AC) (Wu et al., 2016). Following that, the serum expression of the long noncoding RNA GIHCG was evaluated in a total of 46 samples, including 46 ccRCC and 46 AC. The expression of GIHCG was able to differentiate between ccRCC and healthy donors with a sensitivity of 87.0% and a specificity of 84.8%. Particularly remarkable is the fact that it was able to differentiate between early-stage ccRCC and AC (31 stage I ccRCC vs. 46 ACs) with a sensitivity of 80.7% and a specificity of 84.8% (He ZH. et al., 2018).

### 6.3 LncRNAs in autophagy regulation in renal cancer

The lncRNAs can also regulate autophagy in renal cancer. However, only one experiment has evaluated the function of lncRNAs in the modulation of autophagy in the renal cancer. LncRNA HOTAIR is able to sponge miR-17-5p to induce autophagy through Beclin-1 upregulation in the induction of sunitinib resistance (Li D. et al., 2020). Table 1 summarizes the lncRNAs involved in the regulation of urological cancer progression.

**TABLE 1:** The lncRNA-driven regulation of urological cancer progression.

Urological cancer	LncRNA	Remark	References
Prostate cancer	LNC-565686	Increase in the proliferation rate and inhibition of apoptosis via enhancing SND1 stability	Qin et al. (2023)
Prostate cancer	LncRNA TMPO-AS1	Enhancement in the bone metastasis through Wnt upregulation	Wang et al. (2023a)
Prostate cancer	LINC01801	Inhibition of LINC01801 by REST to mediate neuroendocrine differentiation of prostate tumor through autophagy induction	Chang et al. (2023)
Prostate cancer	LncRNA SNHG4	Enhancement in the cell survival and induction of enzalutamide resistance	Dong et al. (2023)
Prostate cancer	LncRNA TYMSOS	Silencing this lncRNA impairs the growth, division and EMT	Xia et al. (2023)
Prostate cancer	TPT1-AS1	Autophagy stimulation to enhance survival	Chen et al. (2024b)
Prostate cancer	CTBP1-AS	Suppressing TP63-induced activation of S100A4	Wu et al. (2024)
Prostate cancer	A1BG-AS1	Transfer by exosomes and reduction in the prostate cancer progression through ZC3H13-induced m6A modification	Yang et al. (2024)
Bladder cancer	LncRNA BCCE4	Increase in the interaction of PD-L1 and PD-1	Zheng et al. (2023)
Bladder cancer	LncRNA AGAP2-AS1	Interaction with IGF2BP2 to enhance tumorigenesis	Zhao et al. (2023)
Bladder cancer	LncRNA-RP11-498C9.13	Antisense lncRNA-RP11-498C9.13 promotes ROS-induced mitophagy to enhance tumorigenesis	Song et al. (2023)
Bladder cancer	LncRNA PVT1	Generating positive feedback loop with STAT5B to increase carcinogenesis	Li et al. (2023b)
Bladder cancer	LncRNA XIST	miR-129-5p/TNFSF10 control to increase cancer progression	Kong et al. (2024)
Bladder cancer	LINC00592	Inducing promoter methylation of WIF1 in carcinogenesis	Wu et al. (2023b)
Bladder cancer	LncRNA MEG	Suppressing EMT through Snail downregulation	Wang et al. (2024b)
Renal cancer	PVT1	Increase in stemness	Wang et al. (2023b)

## 7 Discussion

In the last 10 years, a growing body of research has demonstrated that lncRNAs have a significant role in both the beginning and the advancement of bladder cancer. As of right now, typical biomarkers for bladder cancer are still quite uncommon. This is because they do not possess high sensitivity and specificity, and their use is also rather expensive. There is a need for the development of new biomarkers for the early detection and prognosis of bladder cancer. This is because bladder cancer has a high recurrence rate and a poor prognosis, even after successful transurethral resection and systemic therapy. The purpose of this study is to provide a concise summary of the expression, function, and molecular processes of lncRNAs, as well as the clinical implications of lncRNAs in the diagnosis and prognosis of bladder cancer. There has been research conducted on the molecular processes of lncRNAs in bladder cancer. These mechanisms include lncRNAs interacting with DNA, RNA, and proteins. Both the urine supernatant and the plasma of patients with bladder cancer can be enriched with circulating long noncoding RNAs, which may offer a more favorable potential for developing novel tests for bladder cancer. There is a strong correlation between the abnormal expression of thirty-six lncRNAs and a number of clinical features that are associated with bladder cancer. For the purpose of acting as diagnostic or prognostic markers for breast cancer, the increased lncRNAs offer advantageous traits because of their low expression and less evolutionarily conserved nature. As a result, we investigated thirty lncRNAs that were upregulated in order to identify possible clinical indicators. UCA1 has reasonably good sensitivity, specificity, and area under the curve (AUC), and it may be regarded the most viable diagnostic biomarker for bladder cancer. This is based on the dissection of fifteen upregulated long noncoding RNAs that are connected with the size of the tumor seen in bladder cancer patients. The need of doing large-scale investigations in cells and clinical specimens prior to the development of new lncRNA biomarkers for clinical diagnosis cannot be overstated. In this context, the diagnostic and therapeutic performance of bladder cancer will be facilitated by large and systematic investigations on lncRNAs. The fact that there is now no lncRNA that can be used to the particular diagnosis, prognosis, and therapy of bladder cancer is something that should be taken into consideration. At the same time that microRNAs, circular RNAs, and exosomes all play significant roles in the development of breast cancer, microRNAs are also involved. According to the information that we currently possess, the combination of mRNAs, microRNAs, and lncRNAs would presumably be more effective in improving the early diagnosis and prognosis of bladder cancer (Liu XS. et al., 2021).

Autophagy is indeed a complex biological process that exerts varying effects in different types of tumors, including those within the genitourinary system. In the context of these cancers, autophagy can play dual roles as both a tumor suppressor and promoter, depending on factors such as the specific type of cancer, its stage, and the presence of particular genetic mutations. For instance, in prostate cancer, autophagy has been shown to support tumor cell survival and therapy resistance, particularly in advanced stages where cells experience hypoxic and nutrient-deprived conditions. Conversely, in the early stages, autophagy can suppress tumorigenesis by preventing the accumulation of damaged organelles and proteins, thus maintaining cellular homeostasis. This dual role underscores the need to understand the specific context in which autophagy operates, as it influences treatment strategies and outcomes. Moreover, the regulatory mechanisms of autophagy in genitourinary cancers are influenced by a variety of pathways and molecular interactions, including those involving lncRNAs. For example, in bladder cancer, lncRNAs such as TUG1 and SNHG1 have been implicated in modulating autophagy and contributing to therapy resistance through interactions with key signaling pathways like PI3K/Akt/mTOR and PP2A catalytic subunit, respectively. The complexity is further compounded by the fact that lncRNAs can act as either oncogenes or tumor suppressors, depending on their expression patterns and the regulatory networks they engage with. This variability necessitates a nuanced understanding of the molecular and genetic landscape of each cancer type to effectively target autophagy-related pathways in therapeutic settings. Consequently, more comprehensive studies integrating multi-omics approaches are needed to elucidate these intricate mechanisms and optimize therapeutic strategies targeting autophagy in genitourinary cancers.

Without a shadow of a doubt, long noncoding RNAs play a significant part in the development of several forms of cancer, including rheumatoid cancer, in terms of the biology that underlies the disease, the beginning of cancer, and its spread to distant metastases (Seles et al., 2016). Despite all of the promises and recent breakthroughs in research on lncRNAs, the functional role of lncRNAs is still unknown. lncRNAs have the potential to be connected to a wide variety of physiological and pathological roles, as was previously demonstrated. Nevertheless, phenotypic manifestation and the consequences that it has for the person are of the utmost significance in the end. To research phenotypic expression, it is necessary to alter lncRNAs in order to understand the possible implications of these RNAs. This can be accomplished by a variety of methods, including as the deletion of the promoter region or the whole gene, the incorporation of a premature polyadenylation sequence, antisense oligonucleotide blocking, and other methods (Gutschner et al., 2013; Li and Chang, 2014).

A comprehensive understanding of the role of long non-coding RNAs (lncRNAs) in autophagy, particularly in the context of therapy resistance and urological cancers, necessitates the integration of multi-omics data. By leveraging genomics, transcriptomics, and proteomics, researchers can construct a holistic view of the regulatory networks that underpin the function of lncRNAs. Genomics data provide insights into the genetic variants and mutations that may influence lncRNA expression and function. Identifying single nucleotide polymorphisms (SNPs) and copy number variations (CNVs) associated with lncRNA genes can help in understanding their role in cancer susceptibility and progression. For instance, genomic studies can reveal mutations that disrupt the regulatory elements of lncRNAs, thereby affecting their transcription and subsequent impact on autophagy-related pathways. Transcriptomics data, obtained through RNA sequencing (RNA-seq), offer a detailed landscape of lncRNA expression profiles across different tissues and stages of cancer. This data can identify differentially expressed lncRNAs that are implicated in autophagy. Moreover, transcriptomic analyses can elucidate the co-expression networks between lncRNAs and protein-coding genes, highlighting potential regulatory interactions that govern autophagic processes. Proteomics data, derived from mass spectrometry and other techniques, allow for the quantification and identification of proteins that interact with lncRNAs. These protein-lncRNA interactions are crucial for understanding the mechanistic roles of lncRNAs in autophagy. For example, proteomics can uncover how lncRNAs modulate the activity of key autophagy-related proteins such as Beclin-1 and mTOR. Additionally, proteomic analyses can identify post-translational modifications of proteins that are regulated by lncRNAs, further elucidating their functional roles. Integrating these multi-omics data can reveal the complex regulatory networks involving lncRNAs in autophagy. For example, combining transcriptomic and proteomic data can identify lncRNAs that are co-expressed with autophagy-related genes and their corresponding protein products. Genomic data can then be used to pinpoint genetic variants that influence these regulatory networks. This integrated approach can also aid in the identification of potential biomarkers and therapeutic targets for overcoming therapy resistance in urological cancers. By incorporating multi-omics data, researchers can achieve a more comprehensive understanding of how lncRNAs regulate autophagy, thereby providing new avenues for therapeutic intervention and the development of personalized medicine strategies in urological cancers.

The lncRNAs have emerged as crucial regulators in the development of therapy resistance in various cancers, including prostate, bladder, and renal cancers. These lncRNAs can modulate drug resistance through multiple mechanisms, such as interacting with miRNAs, affecting gene expression at the transcriptional and post-transcriptional levels, and altering signaling pathways. For instance, the lncRNA HOXD-AS1 is upregulated in castration-resistant prostate cancer (CRPC) and interacts with WDR5 to promote the expression of genes involved in cell cycle progression and drug resistance, such as UBE2C, FOXM1, CDC25C, AURKA, and PLK1. This interaction enhances chemotherapy resistance and cell proliferation, making HOXD-AS1 a potential target for overcoming drug resistance in prostate cancer. Another example is the lncRNA NEAT1, which is overexpressed in docetaxel-resistant prostate cancer cells. NEAT1 sponges miR-34a-5p and miR-204-5p, leading to increased expression of ACSL4, which contributes to docetaxel resistance. In bladder cancer, lncRNAs also play significant roles in mediating chemotherapy resistance. The lncRNA TUG1, for instance, is implicated in cisplatin resistance by sponging miR-194-5p and promoting EZH2 expression, which in turn affects cell cycle regulation and apoptosis. Another lncRNA, UCA1, enhances cisplatin and gemcitabine resistance by activating the transcription factor CREB and promoting the expression of miR-196a-5p. In renal cancer, the lncRNA SRLR contributes to sorafenib resistance by interacting with NF-κB and promoting IL-6 transcription, which activates the STAT3 pathway. Similarly, the lncRNA ARSR mediates sunitinib resistance by acting as a competitive endogenous RNA for miR-34 and miR-449, leading to increased levels of AXL and c-MET, which are associated with drug resistance. These examples highlight the diverse mechanisms through which lncRNAs regulate therapy resistance, including modulation of miRNA activity, gene expression, and signaling pathways, making them promising targets for developing novel therapeutic strategies to overcome drug resistance in cancer treatment.

The dual role of autophagy as both a tumor suppressor and promoter is indeed complex and context-dependent. Autophagy can act as a tumor suppressor in the early stages of cancer development by maintaining cellular homeostasis and preventing the accumulation of damaged organelles and proteins, which could lead to genomic instability and oncogenic transformation. In this phase, autophagy helps eliminate potentially malignant cells and suppresses tumor initiation. However, in established tumors, cancer cells can hijack the autophagic process to survive under stressful conditions such as hypoxia, nutrient deprivation, and therapeutic interventions. This switch from tumor-suppressive to tumor-promoting roles of autophagy is influenced by various factors, including the tumor type, stage of cancer, and the cellular microenvironment. Several pathways and mechanisms contribute to this context-dependent switch. For instance, the mTOR pathway, a central regulator of cell growth and metabolism, inhibits autophagy under nutrient-rich conditions, supporting cell growth and proliferation. Conversely, during nutrient starvation, mTOR activity decreases, leading to the induction of autophagy, which can provide metabolic substrates to sustain cancer cell survival and growth. Additionally, hypoxia-inducible factors (HIFs) activated under low oxygen conditions can induce autophagy to adapt to hypoxic stress, thereby promoting tumor progression and resistance to therapy. The involvement of specific lncRNAs in modulating these pathways further underscores the intricate regulation of autophagy in cancer. For example, lncRNA HULC and RHPN1-AS1 have been shown to regulate autophagy and influence therapy resistance in prostate cancer through their interactions with mTOR and EGFR signaling, respectively. Understanding these conditions and mechanisms is crucial for developing targeted therapeutic strategies that can modulate autophagy appropriately depending on the cancer context.

The availability of a number of instances for deletion of lncRNA in cultured cells and animal models, both with and without phenotypic alterations, has recently increased. Neat1, for instance, is a highly abundant long noncoding RNA that is closely related to MALAT1. It is necessary for the development of the mammary glands and the corpus luteum, as well as for the potential of breastfeeding and the creation of pregnancy in mice (Standaert et al., 2014; Nakagawa et al., 2014). On the other hand, knocking out MALAT1 does not appear to have any discernible effects on the pre- and post-natal development of mice (Eißmann et al., 2012; Peters et al., 2016; Zhang et al., 2012; Nakagawa et al., 2012). The deletion of HOTAIR results in surviving mice, but it also causes the spinal vertebrae and metacarpal bones to undergo metamorphosis. On the other hand, the knockout of Fendrr (Foxf1 adjacent non-coding developmental regulatory RNA) leads to embryonic death (Kogure et al., 2013; Sauvageau et al., 2013). Due to the fact that only a small portion of lncRNAs have been studied up until this point, it is not yet feasible to reach a definitive conclusion that explains in full the activities of lncRNAs and their role in physiological and pathological processes. All of the efforts that are being made are ultimately being done with the intention of enhancing the management of cancer in people. To this day, not a single long noncoding RNA has been included into clinical regular practice that is based on urological guidelines (Ljungberg et al., 2015; Babjuk et al., 2013; Hakenberg et al., 2015). However, there are a few candidates that show great promise for treating various forms of cancer (Mouraviev et al., 2016; Chang et al., 2016; Parasramka et al., 2016). In addition, several strategies have been investigated in order to make use of lncRNAs as possible therapeutic agents in the treatment of various forms of cancer. tiny interfering RNAs, ribozymes, aptamers, antisense oligonucleotides, natural antisense transcripts, and tiny compounds are some examples of the methods that fall under this category (Mouraviev et al., 2016; Parasramka et al., 2016). The same may be said for these drugs; they have not yet been included into the standard clinical oncological practice. The use of lncRNAs in RCC is still in its infancy in 2016, with just a few intriguing candidates giving the possibility of application as biomarkers or novel treatment targets. Before the therapeutic use of lncRNAs in patients with RCC becomes a reality, there are still a number of applications and fundamental research investigations that need to be carried out in order to completely understand the underlying processes of their activities.

The role of lncRNAs in regulating autophagy and therapy resistance in urological cancers indeed varies depending on cancer type, stage, and specific genetic mutations. The manuscript discusses how lncRNAs like HULC and RHPN1-AS1 influence autophagy and therapy resistance in prostate cancer by interacting with pathways like mTOR and EGFR signaling. This indicates that lncRNAs can either promote or inhibit autophagy based on their interactions with specific pathways, which can vary depending on the cancer context. For example, HULC promotes survival and resistance to radiotherapy in prostate cancer by upregulating Beclin-1 and downregulating mTOR, while RHPN1-AS1 suppresses autophagy through miR-7-5p sponging and EGFR activation, highlighting the diverse regulatory roles of lncRNAs in autophagy depending on the cellular environment and specific mutations. Additionally, in bladder cancer, lncRNAs like TUG1 and SNHG1 have been shown to modulate autophagy through interactions with signaling pathways such as miR-145/ZEB2 and PP2A catalytic subunit, respectively. The specific impact of these lncRNAs on autophagy and therapy resistance can vary depending on the genetic makeup of the cancer cells and their microenvironment. This context-dependent nature underscores the need for detailed studies to understand the precise conditions under which lncRNAs switch roles from tumor suppression to promotion. Such studies can provide critical insights into how lncRNAs can be targeted for therapeutic interventions, offering a pathway to personalized medicine in treating urological cancers.

The potential of lncRNAs and autophagy-related markers as diagnostic, prognostic, and therapeutic tools in cancer, particularly urological cancers, is promising but indeed requires further validation. As outlined in the manuscript, several lncRNAs, such as HOTAIR, MEG3, and MALAT1, have shown strong correlations with cancer progression, metastasis, and resistance to therapies. For instance, HOTAIR’s involvement in modulating chromatin states and influencing gene expression linked to cancer aggressiveness has been extensively documented, suggesting its potential as a biomarker. However, while preclinical studies and initial clinical observations support their utility, large-scale clinical trials and real-world evidence are necessary to establish their efficacy and safety as clinical biomarkers or therapeutic targets. In particular, the use of lncRNAs as therapeutic targets has been mostly explored in preclinical settings, such as *in vitro* studies and animal models, demonstrating the feasibility of targeting these molecules to modulate autophagy and other cancer-related pathways. For example, the suppression of specific lncRNAs like MALAT1 and HOTAIR has shown to inhibit tumor growth and metastasis in animal models. However, translating these findings into effective clinical interventions requires addressing challenges such as ensuring the specificity and delivery of lncRNA-targeted therapies, minimizing off-target effects, and understanding the complex interactions within the tumor microenvironment. The development of reliable methods for detecting and quantifying lncRNAs in clinical samples is also crucial for their application as biomarkers. Therefore, while the potential clinical applications of lncRNAs and autophagy are compelling, rigorous validation through clinical trials is essential to confirm their utility in improving cancer diagnosis, prognosis, and treatment.

## 8 Conclusion

In order to effectively treat prostate cancer, it is necessary to tailor treatment plans to each individual patient because the disease process is both lengthy and diverse. The molecular processes that are responsible for the pathogenesis of prostate cancer have been gradually revealed as a result of extensive fundamental medical research that has been carried out over the course of the past few years (Smolle et al., 2017). Patients whose condition is resistant to standard anti-hormonal therapy have seen a significant increase in their life expectancy as a result of the introduction of innovative anti-androgens into clinical practice. In the event that certain biomarkers, such as the AR-V7 splice variation in mCRPC, are identified, the treatment may be modified accordingly. LncRNAs are engaged in each and every one of these phases in the growth of the tumor. They could be able to sustain cellular proliferation and invasion independent of androgens, enhance the progression toward castration-resistant states, or preserve androgen-related pathways in the event that androgens are depleted. Some long noncoding RNAs are already being employed as diagnostic biomarkers, while others may be used in the future. Different patterns of lncRNA expression can be used to make prognostic or predictive statements. As therapeutic targets, lncRNAs have the potential to improve the effectiveness of anti-tumor drugs and contribute to the slowing down of the progression of prostate cancer. The method known as RNAi can be utilized to control the production of lncRNAs. Within the framework of this technique, small double-stranded RNAs, such as siRNA, are utilized to cause a degradation of their target lncRNA through the RNA-induced silencing complex (RISC) (Chen et al., 2016c). It is therefore possible to employ the RNA interference technique to successfully lower the expression levels of long noncoding RNAs that have the potential to cause tumors. Antisense oligonucleotides, also known as ASOs, are comprised of either short single-stranded RNAs or DNAs that are antisense to their target long noncoding RNA (Lin et al., 2011). This is yet another approach that may be utilized. Furthermore, the utilization of small molecules has the potential to, for instance, make it impossible for HOTAIR to interact with LSD1 and PRC2 (Chandra Gupta and Nandan Tripathi, 2017; Tsai et al., 2011). It has already been demonstrated that the therapeutic use of the H19-regulated double-stranded DNA plasmid BC-819 has been evaluated and found to be effective in patients who have bladder cancer (Gofrit et al., 2014). The majority of research that has been conducted on the use of lncRNAs as therapeutic targets has been conducted on cell cultures or animal models, and there have been very few studies that have been conducted on human beings. In addition, the precise role of a great number of long noncoding RNAs is still unclear. This is due to the fact that they do not necessarily share a single target or function inside a cell. Furthermore, depending on the kind of tumor, the same lncRNA may perform a variety of other activities. As a result, the utilization of lncRNAs as therapeutic targets may result in unanticipated side effects or significant adverse responses. In spite of this, the more complete our understanding of the role of lncRNAs becomes, the more effective and extensive their therapeutic applications will be. As a result of current study, more long noncoding RNAs that are implicated in the etiology of prostate cancer, as well as their molecular effects and the possible implications for clinical management, will be discovered.

## Glossary

ncRNAs: Non-Coding RNAs
lncRNAs: Long Non-Coding RNAs
mRNAs: Messenger RNAs
miRNAs: MicroRNAs
MVBs: Multivesicular Bodies
CRPC: Castration-Resistant Prostate Cancer
HIFs: Hypoxia-Inducible Factors
EZH2: Enhancer of Zeste Homolog 2
AR: Androgen Receptor
EGFR: Epidermal Growth Factor Receptor
EMT: Epithelial-Mesenchymal Transition
TGFβ1: Transforming Growth Factor Beta 1
mTOR: Mechanistic Target of Rapamycin
PTEN: Phosphatase and Tensin Homolog
VHL: Von Hippel-Lindau
VEGF: Vascular Endothelial Growth Factor
PDGF: Platelet-Derived Growth Factor
HOTAIR: HOX Transcript Antisense RNA
GAS5: Growth Arrest-Specific 5
FOXM1: Forkhead Box M1
SOX2: SRY-Box Transcription Factor 2
HMGA1: High Mobility Group AT-Hook 1
NF-κB: Nuclear Factor Kappa B
AXL: AXL Receptor Tyrosine Kinase
c-MET: Mesenchymal-Epithelial Transition Factor
IL-6: Interleukin 6
STAT3: Signal Transducer and Activator of Transcription 3
UCA1: Urothelial Cancer Associated 1
PCA3: Prostate Cancer Antigen 3
TMPRSS2-ERG: Transmembrane Protease Serine 2 - Erythroblast Transformation-Specific Related Gene
SChLAP1: Second Chromosomal Locus Associated with Prostate Cancer 1
PVT1: Plasmacytoma Variant Translocation 1
MALAT1: Metastasis-Associated Lung Adenocarcinoma Transcript 1
HOXD-AS1: HOXD Antisense Growth-Associated Long Non-Coding RNA 1
NEAT1: Nuclear Enriched Abundant Transcript 1
SOX5: SRY-Box Transcription Factor 5
PRC2: Polycomb Repressive Complex 2
MDM2: Mouse Double Minute 2 Homolog
SND1: Staphylococcal Nuclease and Tudor Domain Containing 1
PTX: Paclitaxel
RCC: Renal Cell Carcinoma
ccRCC: Clear Cell Renal Cell Carcinoma
MNT: Metanephric Tumor
TUG1: Taurine Upregulated Gene 1
CYP4A22: Cytochrome P450 Family 4 Subfamily A Member 22
GHET1: Gastric Carcinoma High Expressed Transcript 1
BLACAT2: Bladder Cancer Associated Transcript 2
SPRY4-IT1: Sprouty 4 Intronic Transcript 1
LINC00857: Long Intergenic Non-Protein Coding RNA 857
TINCR: Terminal Differentiation-Induced Non-Coding RNA
